# Reduction of Survey Sites in Dialectology: A New Methodology Based on Clustering

**DOI:** 10.3389/frai.2021.642505

**Published:** 2021-05-20

**Authors:** Péter Jeszenszky, Carina Steiner, Adrian Leemann

**Affiliations:** Center for the Study of Language and Society, Faculty of Humanities, University of Bern, Bern, Switzerland

**Keywords:** dialectology, survey site selection, subsampling, clustering, language variation and change, dialect survey, linguistic geography

## Abstract

Many language change studies aim for a partial revisitation, i.e., selecting survey sites from previous dialect studies. The central issue of survey site reduction, however, has often been addressed only qualitatively. Cluster analysis offers an innovative means of identifying the most representative survey sites among a set of original survey sites. In this paper, we present a general methodology for finding representative sites for an intended study, potentially applicable to any collection of data about dialects or linguistic variation. We elaborate the quantitative steps of the proposed methodology in the context of the “Linguistic Atlas of Japan” (LAJ). Next, we demonstrate the full application of the methodology on the “Linguistic Atlas of German-speaking Switzerland” (*Germ.:* “Sprachatlas der Deutschen Schweiz”—SDS), with the explicit aim of selecting survey sites corresponding to the aims of the current project “Swiss German Dialects Across Time and Space” (SDATS), which revisits SDS 70 years later. We find that depending on the circumstances and requirements of a study, the proposed methodology, introducing cluster analysis into the survey site reduction process, allows for a greater objectivity in comparison to traditional approaches. We suggest, however, that the suitability of any set of candidate survey sites resulting from the proposed methodology be rigorously revised by experts due to potential incongruences, such as the overlap of objectives and variables across the original and intended studies and ongoing dialect change.

## 1. Introduction

### 1.1. Motivation

Spatial sampling for a dialect study, i.e., choosing localities to survey, has been one of the central issues in dialectology. Similar to the selection of speakers, the selection of surveyed localities (termed “*survey sites”* in this paper) needs careful planning according to study criteria, such as comparability and representativeness of areas, social groups, and linguistic levels. The issue of survey site reduction is as old as surveying itself. Linguistic and, specifically, dialect studies often select their survey sites from earlier data collections, such as linguistic atlases, and choose sites where certain linguistic variables of interest have already been documented. However, site reduction is usually done qualitatively, based on linguistic expertise, without quantitative arguments supporting selection procedures. Thus, despite their importance, most methodologies used for the reduction of survey sites in dialect studies are not reproducible in detail.

Researchers can adopt more objective procedures and potentially optimize their resources by utilizing quantitative methods for locating representative survey sites. Cluster analysis techniques are especially appropriate for this task and are already known in linguistics. This paper outlines a general methodology for the problem of quantitative survey site selection based on previously recorded dialect data (e.g., linguistic atlases) and proposes an application of cluster analysis. To demonstrate the versatility of the approach, the general methodology is also applied to real dialect data sets, in one case with the aim of finding suitable survey sites for an actual contemporary dialect study.

Researchers from different areas of linguistics could potentially benefit from the methodology proposed in this paper, by utilizing survey site networks of previous studies. A potential research aim may be to conduct a dialect interview campaign, revisiting numerous phenomena in a dialect atlas, or recording new phenomena at the same linguistic level as the atlas, with the objective of covering the expected variation in fewer locations. As another example, researchers may want to test how the dialects in a certain area have changed, and so they plan to revisit a previous survey. Assuming that dialect leveling has occurred since the survey, they may only want to visit a sufficient number of sites representing the contemporary dialectal landscape. Additionally, the methodology could be implemented for larger databases based on online crawling or digitized corpora (e.g., Anderwald and Wagner, 2007; Huang et al., 2016; Ueberwasser and Stark, 2017; Grieve et al., 2019; Willis, 2020), where the researcher might need to select limited, representative survey sites after appropriate data pooling (e.g., spatially).

### 1.2. Research Objectives

Linguistic studies often start with the task of survey site selection based on the sites of a previous larger scale survey. Our aim is to provide general suggestions about optimal survey site subsampling to the linguistic/dialectology community. As summarized in the “first law of geography” (Tobler, 1970), variation is assumed to be spatially autocorrelated. Representing variation in linguistic space is therefore deemed to represent the variation within the underlying data in geographic space as well [cf. *Fundamental Dialectological Postulate* (FDP)—Nerbonne and Kleiweg, 2007]. Consequently, subsampling survey sites based on a spatial grid, as often done in dialectology, could theoretically represent linguistic variation. We hypothesize, however, that cluster analysis—already extensively used in dialectometry for finding representative areas and boundaries—can also be utilized for finding representative survey sites for a related or follow-up study.

We address this hypothesis based on two research objectives. First, we propose a general methodology, outlining the steps for finding suitable association measures, subsequent clustering methods and their possible validation, and, finally, a qualitative evaluation of the reduced set of survey sites. Second, we present the practical application of the methodology on the example of the “*Sprachatlas der Deutschen Schweiz*” (SDS—Hotzenköcherle et al., 1962–2003). The specific aim of this application is to reduce the number of survey sites to a representative subset of a predetermined size, to be used for a subsequent study, “Swiss German Dialects Across Time and Space” (SDATS^1^—Leemann et al., 2020c). However, dialect change and socioeconomic processes have occurred since the collection of SDS data (around 1939–1958). This application example includes appointing a candidate survey site subset resulting from the quantitative steps and qualitative revision to estimate *contemporary* dialectal variation, in correspondence to the needs of SDATS. Thus, we address a research requirement beyond finding a representative survey site set in a collection by inferring a future state of language. We argue that most studies aiming to perform survey site reduction have similar objectives and, therefore, would benefit from incorporating these considerations into their methodologies. Additionally, integrated into the outline of the general methodology, we provide a proof of concept based on the “Linguistic Atlas of Japan” (LAJ—NLRI, 1966–1974), demonstrating the breadth of its applicability.

## 2. Background

### 2.1. Site Selection in Spatial Sciences and Dialectology

Finding point-like sampling locations (survey sites) for representing reality is a key issue in spatial sciences, and representativeness is heavily dependent on the spatial structure of the variable of interest. Effective spatial sampling has to consider the spatial autocorrelation in the population, and the variables investigated (e.g., Griffith, 2005; Kumar et al., 2011). Most linguistics surveys focus on multiple variables, necessitating a balanced sampling strategy to capture factors, such as linguistic levels, regional variation of language, and extra-linguistic factors. Practical considerations, such as available respondents and research budgets, impose further constraints on study planning. Linguistic surveys (including large-scale dialect atlases, and projects sampling their sites of interest from previous data sets) often detail their speaker selection criteria (e.g., Linn, 1983) but disclose less about selection process of their survey sites (for exceptions, see MacAulay's review, 2018).

Spatial sciences use numerous sampling strategies (cf. e.g., Ripley, 1981; Olea, 1984; Delmelle, 2009) that are already present in linguistic research. In a *random sampling* approach, each point in a population (or area) has an equal probability of being selected. At the same time, the spatial distribution patterns of linguistic phenomena do not always follow the spatial distribution of other population traits. Therefore, random sampling might lead to oversampling the variable of interest in densely populated regions where few variants prevail, or to undersampling in areas with low, isolated populations that use diverse variants. In linguistics, randomly selecting people has been, however, successfully utilized for sociolinguistic studies, as a large enough sample may be representative of the entire population (Bailey and Dyer, 1992).

*Systematic* or *stratified sampling* divides the population into groups (e.g., Kondo et al., 2014), often by grids in space. Sample sites within this grid (which can be square, hexagonal, adjusted to the population, e.g., by a Voronoi-tessellation, etc.) are chosen systematically or at random. If applied spatially, stratified sampling essentially maximizes the distance between survey sites and gives less chance for undersampling, but might also oversample densely populated areas, where variation may be lower. At the same time, sparsely settled areas may also be oversampled, especially if the variation is lower, for instance, in relatively newly settled or expansion areas of a language (e.g., Western United States, Lapland, Hokkaido, Siberia). *Adjusted sampling* specifically concentrates on avoiding over- and undersampling by densifying the survey site network in areas with higher expected variation (cf. Cressie, 2015). Most traditional large-scale linguistic atlases selected their survey sites based on such spatial grids (cf. McDavid, 1971), e.g., the Slavic Linguistic Atlas (“Obščeslavjanskij lingvističeskij atlas” OLA —Avanesov, 1965), with some regional atlas projects on German dialects using coordinated survey grids (“Sprachatlas von Bayerisch-Schwaben,” SBS—König, 1996–2009; “Vorarlberger Sprachatlas,” VALTS—Gabriel, 1985; “Südwestdeutsche Sprachatlas,” SSA—Steger and Schupp, 1993). A grid method was used for selecting the most central sites of REDE's “Digitaler Wenkeratlas” (DIWA—Lameli et al., 2015) from the original points of the Wenker Atlas. Projects using adjusted sampling include the “Sprach- und Sachatlas Italiens und der Südschweiz” (AIS—Jaberg and Jud, 1928–40), the “Linguistic Atlas of the Middle and Southern Atlantic States” (LAMSAS—Kurath, 1949; McDavid, 1971), and the “New Linguistic Atlas of Japan” (NLJ— Onishi, 2016). SDS is also a relevant example, as sampling was scaled according to linguistic variation over population density.

### 2.2. Grouping and Survey Site Reduction in Dialectology

In a site reduction task, a reduced number of sites are selected from existing samples, such that they are representative of other sites, typically in their neighborhood (cf. Olea, 1984). Computational science provides an extensive coverage of problems related to selecting data points that efficiently describe an entire data set (e.g., Daszykowski et al., 2002; Elhamifar et al., 2012; Gani and Limam, 2016). Spatial sciences (such as soil science and vegetation ecology) and fields where the distribution and change of variables over time are also spatially autocorrelated provide various site reduction methods. For example, Lengyel et al. (2011) select subsets of their vegetation plots by sorting them based on decreasing mean dissimilarity between pairs and then sorted again by increasing variance of these dissimilarities. While many site reduction methods in the spatial sciences focus on finding a subsample for optimizing the extraction of one or a few variables (such as soil attributes, e.g., Maltauro et al., 2019, or species abundance, e.g., Loos et al., 2015), linguistic studies might aim to be representative of tens or hundreds of linguistic variables. Besides, proximity in space *per se* does not define dialect similarity (cf. Szmrecsanyi, 2012), and people, the agents of linguistic variation, are constantly on the move, contributing to a changing spatial distribution of linguistic variables.

Linguistic studies aiming at the comparison of contemporary and older data, however, need to revisit all or a reduced subset of the original survey sites. It is intuitive to convey patterns and trends by grouping sites together for example, by drawing isoglosses and naming dialect areas. According to the law of spatial autocorrelation, nearer sites are expected to be similar and distant ones to be dissimilar (Tobler, 1970; Legendre, 1993; Nerbonne and Kleiweg, 2007). This general correlation is often confirmed in dialectology. Cluster analysis, the quantitative grouping of data, resulting in a lower number of representative groups, is also a fundamental procedure in dialectometry. The general procedure of data analysis in standard modern dialectometry involves the calculation of linguistic distances between every pair of survey sites, producing a linguistic distance matrix. This matrix is then analyzed using a variety of multivariate statistics, including multidimensional scaling and cluster analysis, to identify common patterns of regional variation (Grieve, 2014).

Site reduction can be considered a similar task to finding groups and patterns among survey sites. Most projects that apply site reduction to select sites from earlier collections, usually select their sites, such that they retain the spatial density of sites in the original study (e.g., Séguy, 1973; Kelle, 2001; Bucheli and Glaser, 2002; Lameli et al., 2015; Onishi, 2016; Budin et al., 2019). Spatial autocorrelation is usually assumed without quantitative testing, and the sites are verified case-by-case, introducing potential subjectivity and untested representation. Despite the availability of sophisticated methods for deriving dialect areas and spatial patterns, these methods have not often been used for site reduction.

The methodology presented in this paper fills this research gap, by demonstrating the value of cluster analysis for the task of survey site reduction from previous collections of data.

#### 2.2.1. Cluster Analysis

Most clustering procedures take association matrices (such as linguistic distance matrices) as inputs, based on which clusters are compared (Borcard et al., 2011). There are two relevant clustering techniques important for the methodology in this paper, distinguished by the underlying clustering algorithms, necessary inputs, and analytical procedures. *Hierarchical clustering* is the family of clustering methods mainly used in dialectometry. Its algorithms build a hierarchy among the data points in a nested sequence of partitions (see overviews in Heeringa 2004, p. 146–156; Nerbonne et al. 2008; Levshina 2015, p. 309–311). In hierarchical clustering, every step splits an existing cluster in two, based on a certain metric. Importantly, a linkage criterion is needed to specify the dissimilarity between the clusters present and the newly formed cluster. *Partitional clustering*, usually not used in dialectometry (Nerbonne and Wieling, 2018), aims at breaking the data set into a predetermined number of groups and finds these groups simultaneously, refining the solution in every iteration. Although partitional algorithms disregard hierarchy within the classification, Prokić and Nerbonne (2008) find that the results of the *k*-means partitioning algorithm correspond to dialectal divisions made by experts.

We introduce three clustering methods that are generally considered to perform well in dialectology. According to the arguments of several scholars in dialectology (Heeringa, 2004; Prokić and Nerbonne, 2008; Grieve et al., 2011; Syrjänen et al., 2016; Burridge et al., 2019; Lameli et al., 2020), we decided to test the two most promising hierarchical clustering algorithms and one partitional algorithm. Algorithms in hierarchical clustering differ with regard to their linkage criteria (reviewed in Jain and Dubes, 1988). The *Unweighted Pair Group Method using Arithmetic averages* (UPGMA) method assesses the dissimilarity between the new cluster and the existing cluster based on the distance between their means. In this process, each element in a cluster gets an equal weight, independent of the number of elements in the clusters (Sneath and Sokal, 1973, p. 228). *Ward's algorithm* (1963) works differently with regard to the linkage criterion. It minimizes the within-cluster variance and therefore is prone to producing compact clusters of similar size (within the dimension of linguistic distances) (Wilks, 1995), which is not always reasonable. Grieve et al. (2011) use Ward's method because it is based on the analysis of variance, while Prokić and Nerbonne (2008) find the UPGMA and Ward's method to perform best for dialectometry. Heeringa (2004) provides a comparison between the UPGMA and Ward's method, but finds UPGMA to perform better on Dutch dialect data.

The third algorithm selected is the *Partitioning Around Medoids* (PAM) algorithm (Kaufman and Rousseeuw, 1987), a popular algorithm for clustering non-Euclidean data (Schubert and Rousseeuw, 2019). As a partitioning clustering method, PAM classifies all observations within a data set into *k* number of clusters specified beforehand. The main difference between PAM (also known as *k*-medoids algorithm) and the widely used *k*-means algorithm is that in each step, PAM appoints actual data points (medoids) as the centers of clusters by minimizing the distance between the points and the medoid. *K*-means, however, minimizes the sum of squared Euclidean distances, which makes it less robust to noise and outliers than *k*-medoids (Park and Jun, 2009). Partitioning algorithms are not commonly used in dialectology. However, *k*-means was applied by Hyvönen et al. (2007) and Burridge et al. (2019), while Cheshire et al. (2011) and Syrjänen et al. (2016) applied *k*-medoid clustering on different kinds of linguistic data.

A general problem of clustering procedures is that they always deliver clusters, even if the underlying data has little clustering tendency (e.g., due to dialect continua). Hierarchical clustering is, additionally, prone to large differences in results caused by small changes in the input matrix (cf. Jain and Dubes, 1988; Nerbonne et al., 2008). Therefore, in all cases, clustering procedures need validation in order to obtain stable and interpretable clustering results. Phylogenetic literature (Felsenstein, 2004) and dialectometry (Mucha and Haimerl, 2005; Manni et al., 2006) recommend *bootstrapping*. In dialectometry, bootstrapping resamples a data set with replacement and runs the clustering algorithm for each resampled set, arriving at a “composite” result with information about its stability (Nerbonne et al., 2008). Another popular method, *noisy clustering* builds validation in the clustering procedure by adding noise to the data to test its impact. The advantage of noisy clustering over bootstrapping is that it is also applicable to single distance matrices (Prokić and Nerbonne, 2008). The *cophenetic correlation coefficient* (Sokal and Rohlf, 1962) is often used to measure the correlation between the distances in the original data and the distances as implied by hierarchical clustering results (Heeringa, 2004; Birkenes, 2019). Further internal measures for cluster validation assess the compactness, connectedness, and separation of partitions, including the Dunn-index, which identifies compact (small variance between members) and well-separated clusters (Dunn, 1974).

External evaluation of clustering methods is often undertaken in dialectology through comparing cluster solutions to a gold standard (Heeringa et al., 2002; Prokić and Nerbonne, 2008; Lameli et al., 2020), e.g., to a meticulous qualitative dialect division made by experts. Prokić and Nerbonne (2008) compare the clustering solutions of several algorithms to a benchmark of Bulgarian dialects using the Rand-index (Rand, 1971), entropy, and purity of clusters. This kind of external evaluation may not be available for many potential studies, as the intended number of clusters might not match expert classifications of dialect areas. Meilă's variation of information (*VI*) metric (Meila, 2007), related to the entropy in clusters, compares the similarity of any two clustering partitions, approximating the human intuition of distance.

### 2.3. The Project SDATS

In this paper, we apply the suggested clustering-based site reduction approach suggested to the monumental SDS. This application specifically intends to consider the sociolinguistic aims and other requirements of our project SDATS (Leemann et al., 2020c).

“Swiss German Dialects Across Time and Space” aims at conducting a large-scale collection of the contemporary dialects of Swiss German and a subsequent comparison to dialectal forms recorded in SDS. To reach these goals, SDATS maintains a similar number of participants as SDS (1,000 participants, compared to c. 1,500 in SDS)^2^ recruited from a reduced number of survey sites. Instead of 573 sites in the SDS, SDATS includes 125 survey sites and increases the number of speakers per site to eight speakers (of different social backgrounds) from the 1–3 “Non-mobile Old Rural Males” (and females) recorded in SDS. The main reasons for the site reduction are trends of dialect change in the last 70 years (significant leveling occurred—cf. Christen, 1998), sociolinguistic aims, manpower, and financial resources. Rather than searching for the “base dialect,” as SDS did, SDATS aims to record more intralocal, colloquial variation by interviewing respondents of different backgrounds, with an emphasis on the provenance of respondents. Data collection began in 2020 by means of a custom-developed open-source smartphone application (Leemann et al., 2020b), used mostly in virtual settings (Leemann et al., 2020a) due to the COVID-19 pandemic.

Previous site reduction attempts on SDS have been arbitrary and not replicable. Kelle (2001) digitized 170 SDS maps and selected about one-sixth (101) of the original 573 survey sites, as equidistant as possible, in order to perform a new typological classification and confront traditional qualitative dialect classifications. Unfortunately, his selection criteria are not elaborated (with the exception of equidistance), and site representativeness could not be evaluated without digital data of the whole corpus. Almost in parallel, the “Syntactic Atlas of Swiss German” (SADS—Bucheli and Glaser, 2002) reduced the network of SDS to 383 survey sites. Their selection aimed to keep the comparison of desired isoglosses possible (Glaser and Bart, 2015), and was mainly based on merging villages with a smaller number of inhabitants into single survey sites (Bucheli Berger, 2008).

#### 2.3.1. Digitized SDS Data

Despite being the most comprehensive collection of Swiss German dialect data, SDS has not yet been entirely digitized. Starting in 2007, Yves Scherrer (with the help of his colleagues) undertook the partial digitization of SDS for the sake of several projects; (Scherrer, 2021, 2012; Kellerhals, 2014; Scherrer and Stoeckle, 2016). In Scherrer's process, a subset of variables was defined according to linguistic criteria, with general preference given to phonological and morphosyntactic phenomena. In addition, lexical phenomena that were expected to occur frequently, such as function words, were included (Scherrer, 2021). After scanning and georeferencing the SDS maps, they appointed the locally recorded linguistic variant(s) for each survey site in each map, using geographic information systems. This procedure registered the presence and absence of each variant in digital tables. Scherrer's projects involved a simplified categorization of variables (Scherrer, 2021). This categorization granularity is in many cases (including phonetic variables), not sufficient for SDATS, which aims at a fine-grained comparison across SDS and contemporary dialect usage.

## 3. General Methodology

This section details a general methodology that researchers may consider for a survey site reduction task in order to identify representative sites based on data from a previous, larger-scale dialect study. At each step of the methodology, requirements and possible methods are described, and typical quantitative steps are demonstrated on the example of the LAJ (NLRI, 1966–1974). Then, in section 4, the methodology is applied to SDS data, with the specific goal of appointing survey sites for SDATS.

### 3.1. Requirements and the Steps of the Reduction Process

The general survey site reduction process combines the following quantitative and qualitative steps:

1. Digitize the original database, prepare the linguistic data for the sampling, typically including the (re-)categorization of variants, and select linguistic items appropriate to represent the original data in consideration of the intended study (this step is not explained in detail)
2. Calculate linguistic distance matrices based on the selected linguistic items, thus obtaining association measures among the survey sites, as detailed in section 3.2
3. Carry out the clustering procedures and appoint candidate survey sites in the resulting clusters. Typically, this step involves clustering survey sites based on one or multiple linguistic distance matrices and performing validation tests on clusters. The reduction and the subsequent selection of candidate sites are detailed in sections 3.3 and 3.4
4. Evaluate the candidate survey sites, involving (typically qualitative) revision by dialect experts and through sociogeographic filtering to find sites that correspond to the criteria of the intended study, detailed in section 3.5

To aid researchers potentially implementing this outline in their flow of research, we add a non-exhaustive list of further considerations. Our methodology assumes that the original study is part of a large-scale dialect survey. The correspondence of overlapping items in the intended study and the original data needs to be scrutinized and potentially recategorized. As it appears to be a typical task to infer contemporary dialectal variation from the original data, it is important to select items that are representative at both points in time. Thus, items that are irrelevant for the intended study should be removed, such as names of rural work-tools in a large-scale study of vernaculars. The effect of each variable or groups of variables can be tested by, e.g., jackknifing or other cross-validation methods. The selection of data will always depend on the research question, thus in some cases one or a combination of linguistic levels will be used. Although it is crucial from the point of view of data quality, we do not detail the steps of digitization in this general methodology and we assume that the original data is already digitally available.

The core of the site reduction methodology is a grouping algorithm, which classifies the survey sites within the original database into (a desired amount of) groups, with the aim of finding candidate survey sites in the resulting groups, similar to stratified sampling strategies. As in geospatial analysis, no single resampling strategy is optimal or superior: the method for subsampling also has to be appropriately selected depending on the objectives of the intended study and the original data (cf. Knollová et al., 2005). It is crucial for a researcher to decide what they mean by representativeness when selecting candidate survey sites, e.g., linguistic centrality, spatial centrality, or other, external characteristics. These decisions can be prompted by conducting exploratory analyses on the digitally available data, for example, based on aggregate linguistic distance matrices, visualizing overlaps, or testing clustering tendencies (Lawson and Jurs, 1990).

If the intended study aims to compare findings over time, then selected survey sites should already be present in the original database. Further, beyond the scope of the original data set, the selected survey sites should be representative of the survey sites surrounding them (in a linguistic sense) *at the time of the intended study*. A crucial consideration about the preservation of variation is that site reduction will always eliminate some source of variation, especially with language change occurring since the recording of the original survey. If the goal is capturing diversity, or documenting all linguistic variation possible at the expense of overall representativeness, then field knowledge and qualitative revision are crucial, as even original data or digitized data might not cover all variation. Although the clustering procedure should produce results representative of the original survey, the qualitative evaluation step might overwrite these choices.

#### 3.1.1. The Linguistic Atlas of Japan

Typical quantitative steps in the proposed methodology are demonstrated using data from the LAJ (NLRI, 1966–1974), the largest systematic nationwide dialect collection in Japan. LAJ presents the recorded material of a large-scale survey conducted between 1957 and 1965. In total, 2,400 localities were surveyed across Japan, interviewing one (generally) male speaker per locality, born between 1879 and 1903. The atlas survey contains 285, mostly lexical phenomena.

The data set used in this example contains 37 publicly available^3^ lexical variables from LAJ (Kumagai, 2016), with a focus on basic vocabulary in relation to body parts, weather and time, animals and plants, and levels of kinship. Admittedly, the focus of the data is a risk factor to results being representative of the complete lexical level recorded in LAJ.

To prepare the survey sites for a representative clustering, well-known outliers are removed, leaving 2,238 survey sites. The Ryukyu Islands, in the southwest, are removed due to their large linguistic distance from other parts of Japan. Hokkaido, in the north, was settled by the Japanese primarily as of the end of the 19th century, and thus is removed due to small dialectal variation and mixture.

### 3.2. Linguistic Distance

There should be significant overlap between the original data and the intended study. If that cannot be achieved, a distribution of linguistic data balanced across linguistic levels might be beneficial. Similar to clustering in dialectometry, it is advisable to take as many variables as possible from the original data set, curated for the objectives of the intended study and categorized accordingly.

Once the linguistic basis of the site reduction has been determined, researchers must construct an association measure among the survey sites. In a typical case, a linguistic distance matrix is calculated in a site × site manner, based on a set of linguistic variables.

Methods of linguistic distance calculation vary depending on the linguistic level, the variants' categorization granularity, and, if involved, the details of transcription. For calculating phonetic similarity across variants, edit distances are used most often (cf. Wieling and Nerbonne, 2015). For categorical data, linguistic distance is mostly measured based on presence and absence of variants, e.g., the Hamming distance (Spruit, 2006) or Goebl's (1983) *Relative Identity Value*, calculated on pairwise matches and mismatches. At this point, it would also be possible to test the effect of single variables. Researchers may consider removing variables with spatially similar or correlating distributions as duplicates.

Aggregate linguistic distance matrices can be explored in various ways in order to explore patterns in dialectal variation and to detect outliers and potentially problematic regions. Popular methods include similarity trees, e.g., *Neighbor-net* (cf. Cysouw, 2007), multidimensional scaling (MDS) (Heeringa, 2004; Lameli et al., 2020) (both of which are included in the dialectometry support software Gabmap—Leinonen et al., 2016), or thematic maps. The latter may focus on one certain survey site or present the aggregate picture in linguistic distance maps (Goebl, 1982; Scherrer and Stoeckle, 2016). Such plots and maps, in essence, help discover clustering tendencies and gradual transitions among dialect areas (based on the limited data).

#### 3.2.1. Linguistic Distance Calculation Applied to LAJ

For LAJ, the linguistic distance matrix is calculated using a formula based on Goebl's *Relative Identity Value* (*RIV*_*jk*_) (1983), similar to Scherrer and Stoeckle (2016) as applied in Jeszenszky et al. (2019). For each lexical variable, the variants (up to hundreds in some cases) are categorized on two levels. First, variant categories are constructed based on phonetic similarity. Within variant categories, further distinction is made between individual variants: variants within a variant category receive a flat difference rate^4^.

We use an MDS approach to discover latent clusters and dialect continua in the data. We plot the first two or three dimensions of the multidimensional scaling results and associate the first three dimensions to RGB colors and map them^5^. These visualizations show that continua are present in this data set, thus clusters with lower stability and more (spatial) overlap are expected.

### 3.3. Clustering

The linguistic distance matrix is the input of clustering algorithms used for site reduction. Dialectometry often uses cluster analysis to find the internally most homogeneous and externally most heterogeneous groups in dialect data. Importantly, however, clustering techniques have mostly been used to find the optimal split^6^ and spatial distribution in the data, thereby often defining dialect areas. In a typical site reduction study, however, the researcher would aim for much more than the optimal number of clusters in the data.

Hierarchical clustering results in dendrograms and association values between survey sites. Dendrograms cut at the desired or optimized level can also be spatially represented by a cluster map. Partitional clustering produces a predetermined number of clusters, the optimal number of which can be determined by optimization. Researchers might not know the exact number of survey sites they want to extract from the original set, which might influence the choice of clustering method. In any case, it is worth experimenting with different numbers of clusters, also around a previously decided number, in particular for exploratory analyses. Depending on their aims, researchers might determine *k* clusters directly for a partitional method, or, for a hierarchical method, they might select a cophenetic distance, beneath which they find their *k* clusters in the dendrogram. It is always possible to adjust the final number of survey sites in a qualitative revision.

#### 3.3.1. Application of Clustering to LAJ Data

We demonstrate the performance of three clustering algorithms (PAM, UPGMA, and Ward's method), using the example of LAJ. All three clustering algorithms are implemented using the fpc package (Hennig, 2020)^7^ in R (R Core Team, 2020). We perform clustering on the linguistic distance matrix resulting from section 3.2.1. Using each clustering method, partitions of *k* = 20, 50, 100, 150, 200, 300, 400, and 500 clusters are produced.

To validate the results of the different clustering methods, we used a bootstrapping approach (e.g., Nerbonne et al., 2008), as included in the fpc package. In the bootstrapping approach, each cluster is calculated in 100 bootstraps (default value) with resampled data. For each cluster, the Jaccard-similarities of the initial cluster solution (in bootstrap nr. 1) to cluster solutions in all other bootstraps are computed (Hennig, 2007). This approach provides *stability* values for each “composite” cluster found, based on which the performance of clustering algorithms and the sensibility of the choice of *k* (number of clusters) can be assessed.

**Figure 1:**
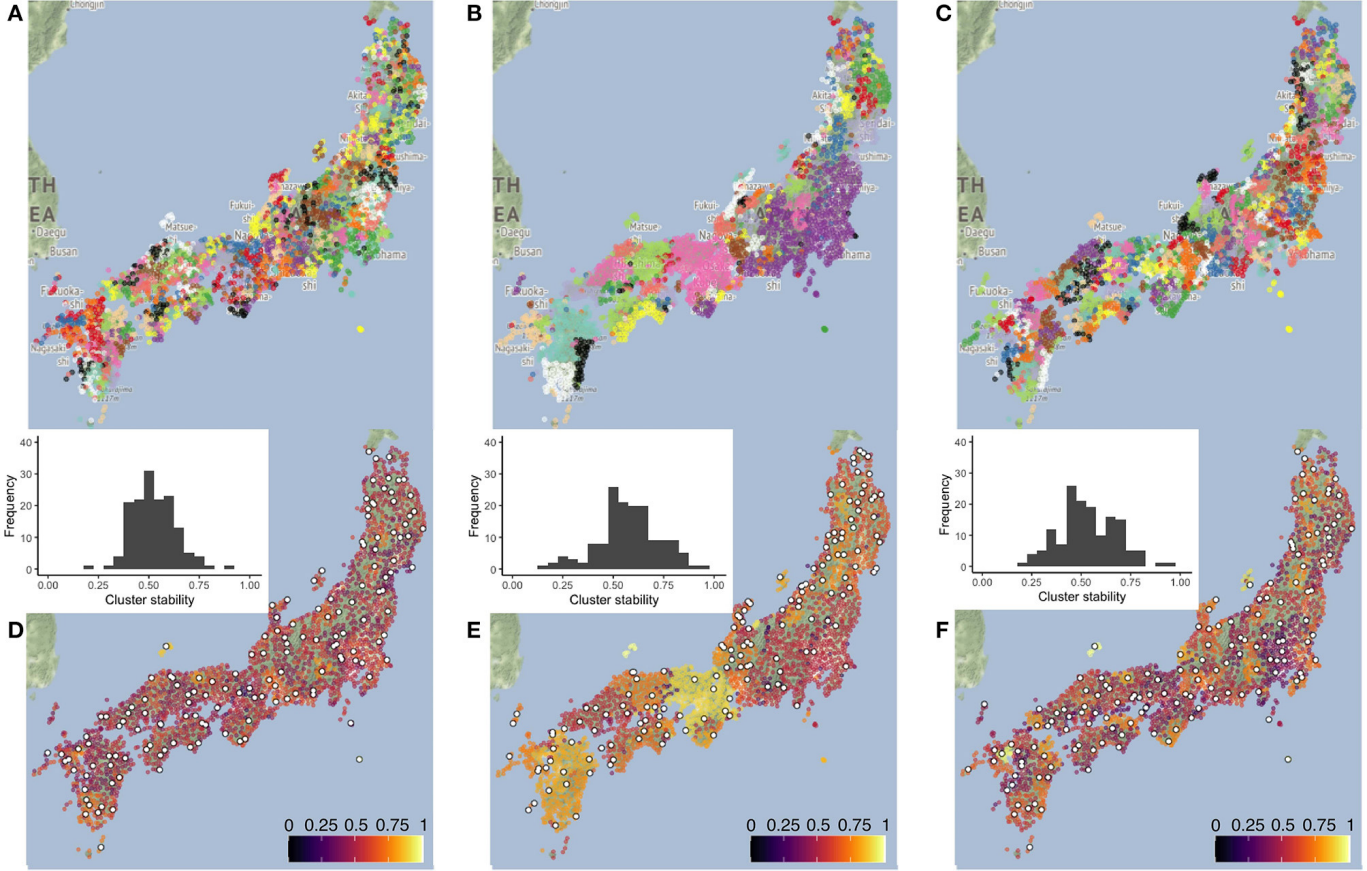
The cluster maps **(A–C)**, stability maps **(D–F)**, and the stability histograms for the three clustering methods PAM (left), UPGMA (center), and Ward's (right) method, calculated for *k* = 150 clusters. Clusters in maps **(A–C)** are presented on a diverging scale of 15 colors, which repeat. Stability maps **(D–F)** also contain candidate survey sites selected based on linguistic centrality within their own cluster. Large differences are visible across the cluster solutions and smaller differences across the stability of the clusters. Besides, stability shows little spatial autocorrelation beyond the large size of clusters found by UPGMA.

As linguistic variation is assumed to be spatially autocorrelated, members of clusters found in the data are supposed to be clustered in space as well. To confirm this, and to visually explore the spatial patterns, we map the clusters produced, along with their stability values. Figure 1 presents the clusters found in the database by the three clustering algorithms (Maps A—PAM, B—UPGMA, and C—Ward's method) with *k* = 150, an overall large number for site reduction requirements given the number of sites in LAJ. Clusters are presented on a diverging, repeating color scale. In Maps D–F, the cluster stability values are mapped to the members of the clusters. Such stability values should not be evaluated solely based on descriptive statistics (e.g., means and standard deviation) as they may vary substantially across clusters, further justifying mapping. Maps D–F also contain the histograms of the stability values, presenting considerable deviations from a normal distribution.

In PAM's map (A), clusters do not appear spatially compact, although members are clustered in the same region. In UPGMA's map (B), several clusters are visible with a high number of members. UPGMA is based on the average difference between clusters and, because of this, chaining effects are not typical for this algorithm (Lameli et al., 2020). The small, unstable clusters of single members (*singletons*) are thus possibly outliers in the linguistic space, found as clusters by this method. UPGMA shows 39 singletons for *k* = 150 while the other methods show none. Ward's method, based on their positions in the map (C) seems to find clusters structurally more similar to UPGMA. Compact clusters are a characteristic of Ward's method, contributing to its popularity in dialectology. The clusters in Map F look somewhat more stable and spatially more compact than those in PAM, and clusters of the same color (Map C) present clearer boundaries in space, appearing to overlap less. Based on the stability histograms in D–F, PAM seems to have the lowest overall values, making it less suitable for clustering than the hierarchical methods (in case of *k* = 150). It is intuitive to expect lower stability when *k* is higher, as smaller clusters are expected to be also more similar to neighboring clusters. This is even more significant when (dialectal) continua are present in the data, as in the case of Japanese. When applying multiple clustering algorithms with different *k*, it is interesting to see which algorithm produces more stable results with a certain number of clusters.

In terms of external validation,^8^ Meilă's *VI* (Meila, 2007) can also be used for calculating similarities across clustering solutions. In Table 1, we compare the clustering solutions resulting from the three clustering methods with different *k* number of clusters. Higher values of Meilă's *VI* indicate greater variation between cluster solutions. Larger *k* usually means larger potential difference, but in our case *k* grows to a degree where difference between cluster solutions cannot increase anymore. Indeed, above *k* = 150, *VI* values start to decrease again. Table 1 shows, somewhat surprisingly, that PAM is more different from the two other solutions than they are from each other, despite UPGMA's tendency for producing large clusters and singletons. This might mean that UPGMA's clusters contain, or are structurally similar to Ward's clusters, while PAM's clusters might not overlap well with Ward's. PAM's lower stability and UPGMA's and Ward's method's different cluster solutions, despite their structural similarity, suggests that researchers should strongly consider the choice of cluster algorithms.

**Table 1:** Meilă's *VI* values, comparing the cluster partitions across the three bootstrapped clustering solutions for *k* = 20, 50, 100, 150, 200, 300, 400, and 500.

	***k***	**UPGMA**	**Ward**		***k***	**UPGMA**	**Ward**
**PAM**	20	1.9338	2.0656	**PAM**	200	2.4114	2.3403
**UPGMA**	20		1.4823	**UPGMA**	200		1.8859
**PAM**	50	2.1974	2.2349	**PAM**	300	2.1907	2.0897
**UPGMA**	50		1.6984	**UPGMA**	300		1.7146
**PAM**	100	2.4787	2.3981	**PAM**	400	2.0009	1.8902
**UPGMA**	100		1.9108	**UPGMA**	400		1.5331
**PAM**	150	2.5056	2.4467	**PAM**	500	1.8243	1.7581
**UPGMA**	150		1.9315	**UPGMA**	500		1.3471

*The higher Meilă's VI, the more different the cluster partitions are*.

### 3.4. Selecting Candidate Survey Sites

Once the validity and stability of clusters are assessed, representative sites can be identified. This can be regarded as an analogy to stratified sampling strategy, where one point is selected from each stratum. In our case, strata are the clusters, the partitions in the abstract linguistic dimensions. Studies might differ in terms of requirements for representative sites, generating several methodological considerations.

Studies might differ in the distribution granularity of variants. Resampling has to consider this granularity, along with other spatial patterns. If capturing fine-grained spatial variation is the aim, then sampling density should be adjusted accordingly. One approach could be choosing points in clusters that are central in a linguistic sense. This approach is demonstrated on LAJ in section 3.4.1 and on SDS in section 4.2.2. In case of running multiple clustering procedures and composite dendrograms, the representative sites' identity becomes less obvious, as clusters from different runs overlap. It is possible, however, to appoint a central site for each calculated cluster and count the number of times each survey site becomes the central one. This approach is implemented in section 4.3.

Depending on the time elapsed since the original data collection, it might also be useful to estimate dialect change. One approach is to assume that “linguistic gravity” (Trudgill, 1974) has driven local varieties to become more similar to, e.g., the most populous nearby survey site. In this sense, linguistic gravity can be used to estimate language change emanating from local hubs into their *hinterlands*, making them more similar to the hub. Such patterns are often associated with dialect leveling, e.g., in Swiss German dialects (cf. Christen, 1998). This approach is also implemented on SDS in section 4.2.2 and section 4.3.2.

Geography playing a small role in selecting candidate sites is relatively small, as clustering happens in the linguistic dimensions. It is, nevertheless, intuitive to designate the spatially central point in a cluster as a candidate, and surveys in dialectology often set out from equidistant samples, based on thorough qualitative arguments. For example, in case of limited or biased available data, this strategy may be reasonable for the estimation of a hypothetical future linguistically central point.

Beyond these aspects and the objectives of the intended study, candidate survey sites might also be selected using external characteristics of the survey sites or a ranked eligibility measure of multiple characteristics. In case of studies interested in smaller areas or a few survey sites, qualitative methods may suffice from this point onward. If stable clusters are obtained, it is possible to investigate them one by one to choose the sites most appropriate for the contemporary dialectal variation and the intended study.

#### 3.4.1. Candidate Selection in LAJ

For LAJ, we find linguistically central survey sites in each cluster by summing linguistic distances within clusters. The survey site with the smallest total linguistic distance within the cluster becomes the candidate site. In case of PAM, this point is exactly the *medoid*. Figure 1 plots, for *k* = 150, the candidate sites for each clustering method in Figures 1D–F, as white points with a black contour. Candidate sites from PAM and Ward's method are identical in 63 cases, whereas their overlap with UPGMA is much lower (21 and 9, respectively).

Candidate sites in the case of UPGMA are distributed more evenly than the cluster structure, comprising several large clusters, suggests. This is due to singletons and unlikely clusters that are made up of several sites farther apart (such as the blue sites in Map B scattered within the largest purple cluster in the east—around Tokyo). Accepting these candidate sites as a reduced set of survey sites would cause problems in representation of spatially surrounding dialects.

The continuous nature of the data and the validity of FDP are confirmed by Jeszenszky et al. (2019). Based on the cluster structure, cluster stability patterns, and the patterns of candidate sites seen in Figure 1, we conclude that Ward's method is the most well-grounded for *k* = 150. Due to their spatially compact clusters it yields, Ward's method presents itself as the safest bet, knowing the bias in the data because of the phenomena it contains. In contrast, UPGMA produces unrealistically large dialect areas and unreasonable singletons, and PAM is less stable with more clusters overlapping in space.

### 3.5. Evaluation and Revision of Candidate Survey Sites

The candidate survey site sets resulting from the site reduction procedures are assumed to be representative of the original data. However, their main aim, as candidates, is to provide a quantitatively supported starting point for determining the sites that actually need to be researched. Several reasons call for a further qualitative evaluation of candidate survey sites. First, the linguistic basis of the site reduction might not be perfect due to various potential factors within the original database, the requirements of the intended study, and the circumstances that might have changed since the original survey. Second, dialect change may have progressed, due to people's changing way of life, mobility patterns, language attitudes, etc. Third, potential survey sites might have changed with regard to their sociodemographic settings, language policies, etc. Therefore, any set of candidate survey sites has to be revised in accordance with the requirements of the intended research, which potentially collects contemporary dialect data. Generally, the potential uncertainty about representativeness of contemporary dialectal variation increases with time elapsed since the original data was recorded, thus increasing the value of expert revision. Depending on the study's aims, the step of evaluation may result in swapping sites, adding sites that were originally not recorded, selecting more than one site from a cluster, rebalancing a clustering solution based on a spatial grid, etc. In section 4.4, we provide a qualitative revision of a candidate site set from SDS.

## 4. Application Example: SDATS

In this real-life example, we present the entire site reduction procedure as applied to digital data from the “Sprachatlas der deutschen Schweiz” (SDS), with the aim of finding survey sites corresponding to the requirements of the contemporary dialect research project SDATS. Thus, the final goal is to find a way to represent the estimated contemporary variation, inferred from the original data and revised based on experts' field knowledge.

“Swiss German Dialects Across Time and Space” aims for candidate sites that are linguistically as different from one another as possible, thereby covering the largest swath of dialectal forms used. We carry out the clustering experiment with two different approaches on the same data set. First, Approach I is used for the demonstration of a generalizable methodology, presented in section 4.2. This approach applies the quantitative steps of the methodology similarly to the example in section 3. Second, Approach II is used to arrive at the survey sites actually used in SDATS, as detailed in section 4.3. This approach applies only the PAM clustering algorithm with a different custom-made validation approach. Then, candidate survey sites are revised to represent the contemporary dialectal variation, in section 4.4.

### 4.1. Linguistic Distance

Scherrer's digitized SDS database (termed Scherrer's data)^9^ covers 289 linguistic variables: 107 phonetic, 118 morphosyntactic, and 64 lexical variables (Scherrer, 2021). SDATS's initial plans included revisiting 200 linguistic phenomena in SDS. At the time of selecting the survey sites, however, the extent of the overlap of SDATS variables with Scherrer's data was not clear yet, therefore all digitized variables were utilized for the site reduction.

The linguistic distance matrix is calculated similarly to section 3.2.1, based on Goebl's *Relative Identity Value* (*RIV*_*jk*_) (Goebl, 1983; Jeszenszky et al., 2019). For each variable, the difference based on the variant categories is noted for each survey site pair, allowing for multiple answers. The final linguistic distance between a survey site pair is the proportion of the differing variables among those variables where an answer is present for both survey sites (*n*), or

(1) $$\begin{array}{r} D_{ij}^{ling}=\frac{\sum D_{Q}}{n} \end{array}$$

where *D*_*Q*_ is the number of diverging variables regarding survey sites *i* and *j*. For example, if in survey sites *i* and *j* answers for all linguistic variables are in different variant categories, then a linguistic distance of 1 is assigned to this survey site pair^10^. To discover linguistic distances in an aggregate manner, it is possible to use multidimensional scaling and thematic mapping^11^.

### 4.2. Site Reduction: Approach I—Bootstrap Clustering

“Swiss German Dialects Across Time and Space” aims to select 125 survey sites and has the objective of collecting a balanced set of phenomena across linguistic levels Therefore, we intend to use data from SDS such that is also balanced across the linguistic levels. We group the linguistic variables according to the linguistic levels and calculate the linguistic distance matrices for each of them. To counter the higher numbers of morphosyntactic and phonetic variables, the mean value of these three matrices (termed the *mean linguistic distance matrix*, $-\bar{LD}$) is the input for the clustering steps. Note, however, that doing so leads to the increased weight of individual lexical phenomena.

We apply the three clustering methods presented in section 3.3.1. We perform clustering with bootstrapping on $\bar{LD}$ using PAM, UPGMA, and Ward's method from the fpc package, with *k* = 125, in accordance with the hard criterion in SDATS. Similar to section 3.3.1, we use the stability values associated with clusters as the method of internal validation. We also calculate Meilă's *VI* to compare clustering solutions' similarity across methods and across clustering solutions resulting from different subsets of the data. In addition, in section 4.2.2, we test how well different sets of candidate survey sites represent the original $\bar{LD}$.

#### 4.2.1. Clustering and Validation

**Figure 2:**
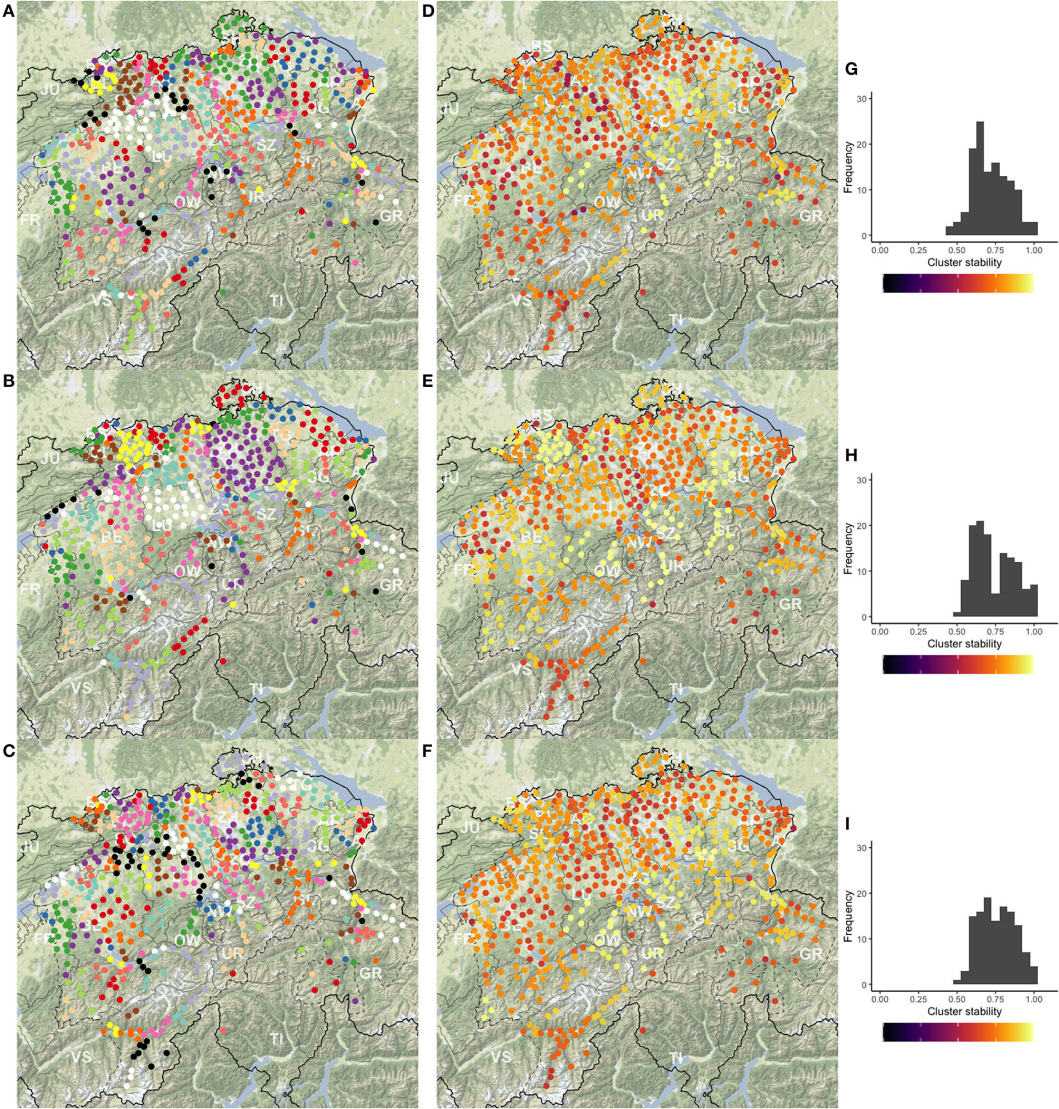
The cluster maps **(A–C)**, stability maps **(D–F)**, and the stability histograms **(G–I)** for the three clustering methods PAM (top), UPGMA (center), and Ward's (bottom) method, calculated for *k* = 125 clusters. Clusters in maps **(A–C)** are presented on a diverging scale of 15 colors, which repeat. Substantial differences are visible across the cluster solutions and smaller differences across the stability of the clusters across the methods. Importantly, clusters in some regions stay stable independent of the clustering method.

Figure 2 maps cluster solutions based on the three clustering methods, PAM (A), UPGMA (B), and Ward's method (C). As expected, cluster members are also spatially clustered in the overwhelming majority of the cases. In a few cases, members of a cluster are separated by members of other clusters. In addition, singletons are present. Both PAM's and Ward's maps show spatially compact clusters (corresponding to the FDP), while the UPGMA map is more prone to producing larger clusters and clusters of singleton outliers. UPGMA finds 50 singletons, while PAM and Ward's method find 27 and 17, respectively. These patterns are structurally similar to the clustering results of LAJ.

Figures 2D–F present the stability of clusters. It is visible, here, that some clusters are stable regardless of the clustering method, while values vary in other areas. Most of the Swiss Plateau^12^ shows low cluster stability, especially with PAM. Overall stable regions include the cantons of Schwyz (SZ), Uri (UR), Obwalden (OW), Glarus (GL), the Entlebuch region in the canton of Lucerne (LU), the Haslital region and the SE part of the Bernese Oberland in canton Berne (BE), and the Eschenbach region of canton St. Gallen (SG). The singleton survey sites, e.g., in the canton of Graubünden (GR) and elsewhere do not show very high stability, independent of clustering method. Interestingly, UPGMA and Ward's method provide stable clusters in the canton of Basle-Country (BL), while PAM and Ward's method show stability in the Oberland region of canton Zurich (ZH).

Stability values are also presented as histograms in Figures 2G–I. The skew toward the right implies that the bulk of clusters are stable, with little difference between the clustering methods. Based on the stability values, the cluster structures and the field expertise of SDATS project members, each cluster solution is deemed acceptable for the production of *candidate* sites. However, there are some evident drawbacks. PAM's stability, on average, seems lower, but the differences in the maps and histograms are visually not as substantial as those seen in the application to LAJ data. UPGMA's larger clusters and singletons are often linguistically not supported (e.g., an expert would expect to find more clusters in the canton of Valais—VS). Finally, the compact and similar-sized clusters of Ward's method are tempting for dialectology, but they are often unreasonable, e.g., in the Swiss Plateau.

#### 4.2.2. Selection of Candidate Survey Sites

The next step in the methodology is appointing a candidate survey site within each cluster. We can select linguistically central sites, defined by the smallest total linguistic distance toward cluster members. Appointing this site intrinsically makes PAM a practical method for the application. However, as SDATS aims to investigate contemporary dialectal variation, we select candidate survey sited based on estimated potential dialectal change since data collection in SDS. We aim to find sites that have potentially influenced their local surroundings since 1950, assuming, based on Trudgill's linguistic gravity theory (1974), their surroundings have become more similar to them (Christen, 1998; Szmrecsanyi, 2012; Schmid et al., 2019). To address this, we select survey sites with the highest population in 2018 from each cluster, using official census data (BFS, 2018).

**Figure 3:**
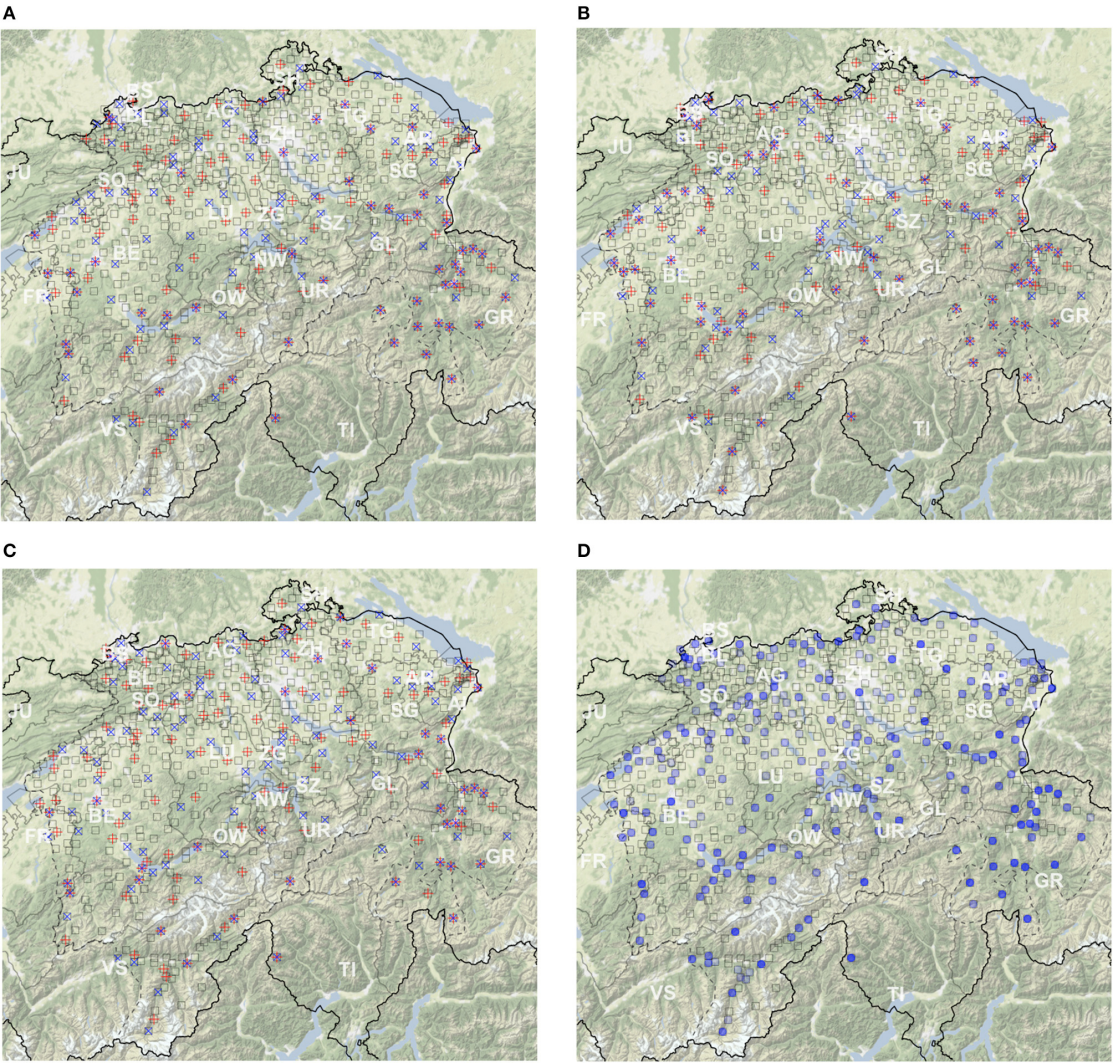
Maps of the two types of candidate survey sites. Centers are appointed by the smallest total linguistic distance within a cluster (red +), and the highest population within a cluster (blue ×), for each clustering method (**A**—PAM; **B**—UPGMA; and **C**—Ward's method). Map **(D)** shows all candidate survey sites in a stacked manner. All maps contain the original SDS survey sites in the background (gray squares).

Figure 3 presents these two kinds of candidate survey sites sets for the three clustering methods (A—PAM; B—UPGMA; and C—Ward's). Linguistically central sites are depicted by +'s, and sites with the highest population by ×'s. In Map D, all candidate sites from the other three maps are stacked, to show the potential eligibility of any SDS survey site. In the case of UPGMA (Map B), the two requirements overlap in more than half of the cases, though this happens less frequently for the other two clustering methods. Maps A–C convey the message that the site with the highest population might not be the linguistically central or representative site with regard to the original data, suggesting that estimating future linguistic scenarios based on linguistic gravity should be approached with caution. In Map D, overlaps of the symbols show a higher potential eligibility of sites in the Alps, especially in the canton of Graubünden (GR), with the latter due to the high proportion of singleton clusters. This, nevertheless, hints at the presence of unique dialects.

To evaluate these candidate site sets, we test if they are representative of the original survey site set of SDS (573 sites). Technically, we test if the similarity of the distributions in the linguistic distance matrices of the candidate set and the SDS set ($\bar{LD}$) is statistically significant. Since the values in the matrices of the original set and in the candidate sets are not normally distributed, we use the *Kruskal—Wallis test* to test the significance of the differences. Affirming this, the *pairwise Wilcoxon rank sum test* allows us to test which candidate sets' linguistic distance matrices have a significantly different distribution from the original $\bar{LD}$. In addition to the candidate sets, we test the performance of random site sets as well. For each of the clustering methods, we create ten random site sets, selecting one random site from each of their clusters. Further, we create 1,000 unrestricted random site sets from the SDS survey sites.

The Wilcoxon rank sum tests shows that no candidate site set presents a significant difference from the original linguistic matrix set. Importantly, however, only 27.64% of the unrestricted random sets show a significant difference from $\bar{LD}$. This value is still 40.7% when sinking *p*-value's threshold to 0.001. At the same time, random site sets from clusters never exhibit a *p*-value over 5 × 10^−13^. Thus, there is substantial possibility that an unrestricted random sample becomes representative of the whole population. We argue that this is due to sampling one out of five points, a relatively large sample, and that a threshold of representativeness has to be cautiously applied by the researcher.

**Figure 4:**
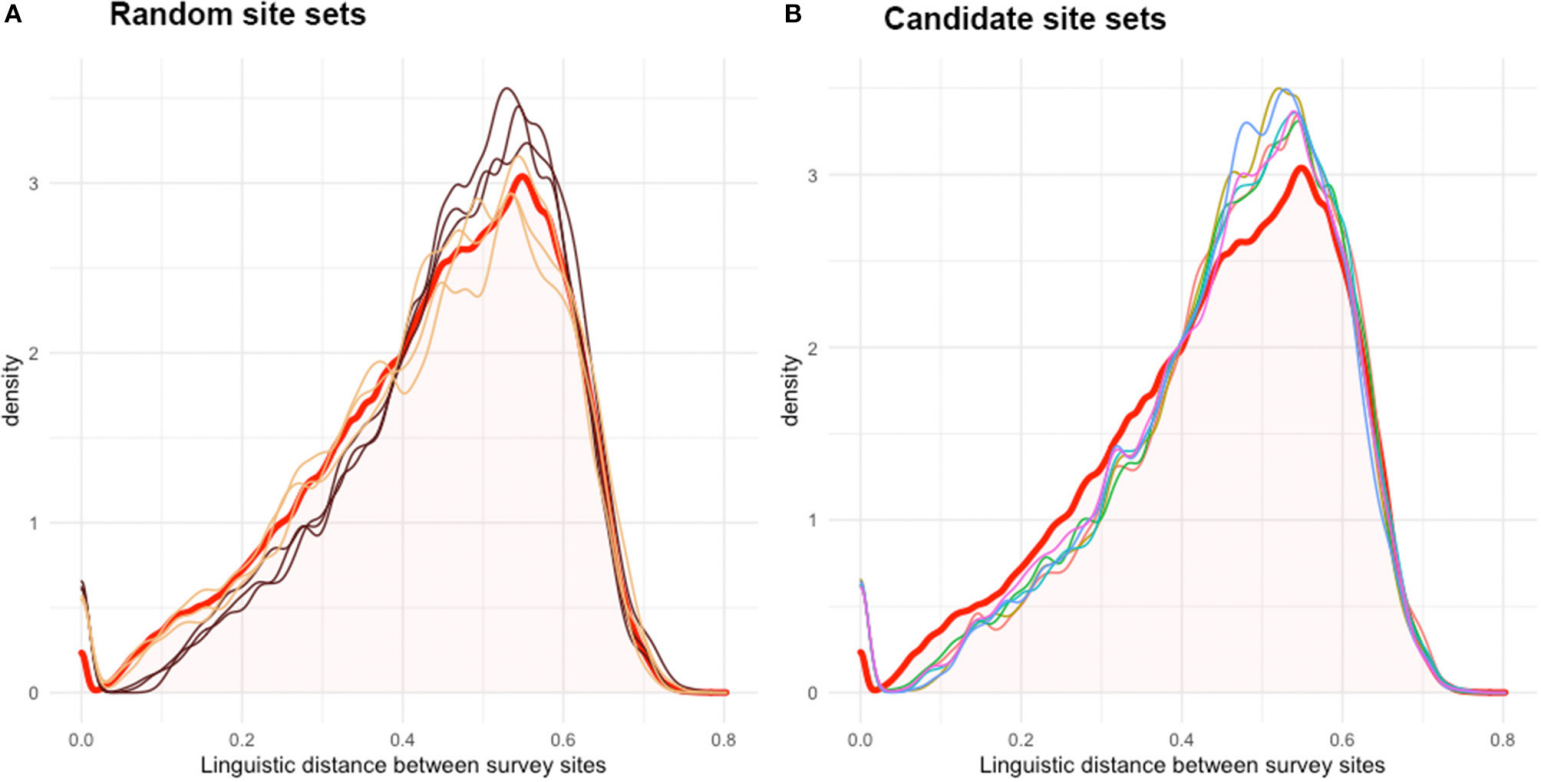
Density graph of the values within linguistic distance matrices. In **(A)**, the linguistic distance matrices from three unrestricted random sets (yellow) and random sets from the resulting clusters of each clustering method (brown) are plotted together with the original mean linguistic distance matrix ($\bar{LD}$—red). In **(B)**, linguistic distance matrices from the candidate survey site sets are presented. Beside unrestricted random sets, all sets overshoot the peak density of $\bar{LD}$. Thus, on average, linguistic distances are larger within these candidate site sets, as expected, and their members are linguistically more different from one another.

Figure 4 presents some distributions of the linguistic distance matrices of the candidate site sets in relation to the original $\bar{LD}$. Figure 4A presents six random sets from the previous test, with random sets from clusters in brown and unrestricted random sets in yellow. Brown lines stay below the distribution of $\bar{LD}$ in the left side and overshoot $\bar{LD}$ at its peak. Yellow lines follow the distribution of $\bar{LD}$ more exactly, but this is not always enough to be representative. Figure 4B presents the densities of the candidate site sets. All lines stay somewhat below the distribution of $\bar{LD}$ on the left side and overshoot the $\bar{LD}$ at its peak. This means that the candidate survey site sets include more of those survey sites that have a higher linguistic distance toward one another, ultimately intensifying the variation present in the candidate set while overlooking survey sites that are less diverse, thus less different from one another.

### 4.3. Site Reduction: Approach II—Candidates Resulting From Ranking

This section serves the purpose of detailing the site reduction approach implemented to define the conclusive candidate site set for SDATS, which is used from section 4.4 for the qualitative revision. We present a customized methodology of cluster analysis and site selection to fulfill two aims. First, we address SDATS' requirement of balance across linguistic levels. Second, we address the requirement of inferring a future linguistic situation based on the theory of linguistic gravity. To this end, we use a special-purpose cluster validation technique and build the qualitative requirements of the SDATS project partly into the clustering step (i.e., selecting a candidate site with a relatively high population from a cluster). We arrive at the candidate survey site set by ranking the survey sites based on two measures introduced below, one related to their stability in their clusters (*J*), and another based on their population (*P*_*top*_).

Scherrer's data are imbalanced across linguistic levels and it contain 107 phonetic, 118 morphosyntactic, and 64 lexical items. In section 4.2, we calculated the mean linguistic distance based on the three linguistic levels. This means, however, that the weight of each lexical item is almost double the morphosyntactic items. The approach introduced here is proposed as an experimental method to counter this effect. In order to get a sample of items representative of each linguistic level, we create *S* subsets, drawing equal numbers of random items from each of the three linguistic levels. On the one hand, we randomly select 64 items from each linguistic level (referred to as subsets *S*^64^)^13^. On the other hand, we randomly select 20 items from each linguistic level (referred to as subsets *S*^20^). The number 64 is decided by the number of lexical items in Scherrer's data, the lowest among the linguistic levels. In parallel, sets of 20 items are used to decrease the bias assumed to be caused by the constant presence of all 64 lexical items in the *S*^64^ subsets. We create 35 subsets of *S*^20^ ($S_{101}^{20}$, $S_{102}^{20}$, $S_{103}^{20}$ ... $S_{135}^{20}$), and 30 subsets of *S*^64^ ($S_{1}^{64}$, $S_{2}^{64}$ ... $S_{5}^{64}$, and $S_{201}^{64}$, $S_{202}^{64}$, $S_{203}^{64}$ ... $S_{225}^{64}$)^14^.

**Figure 5:**
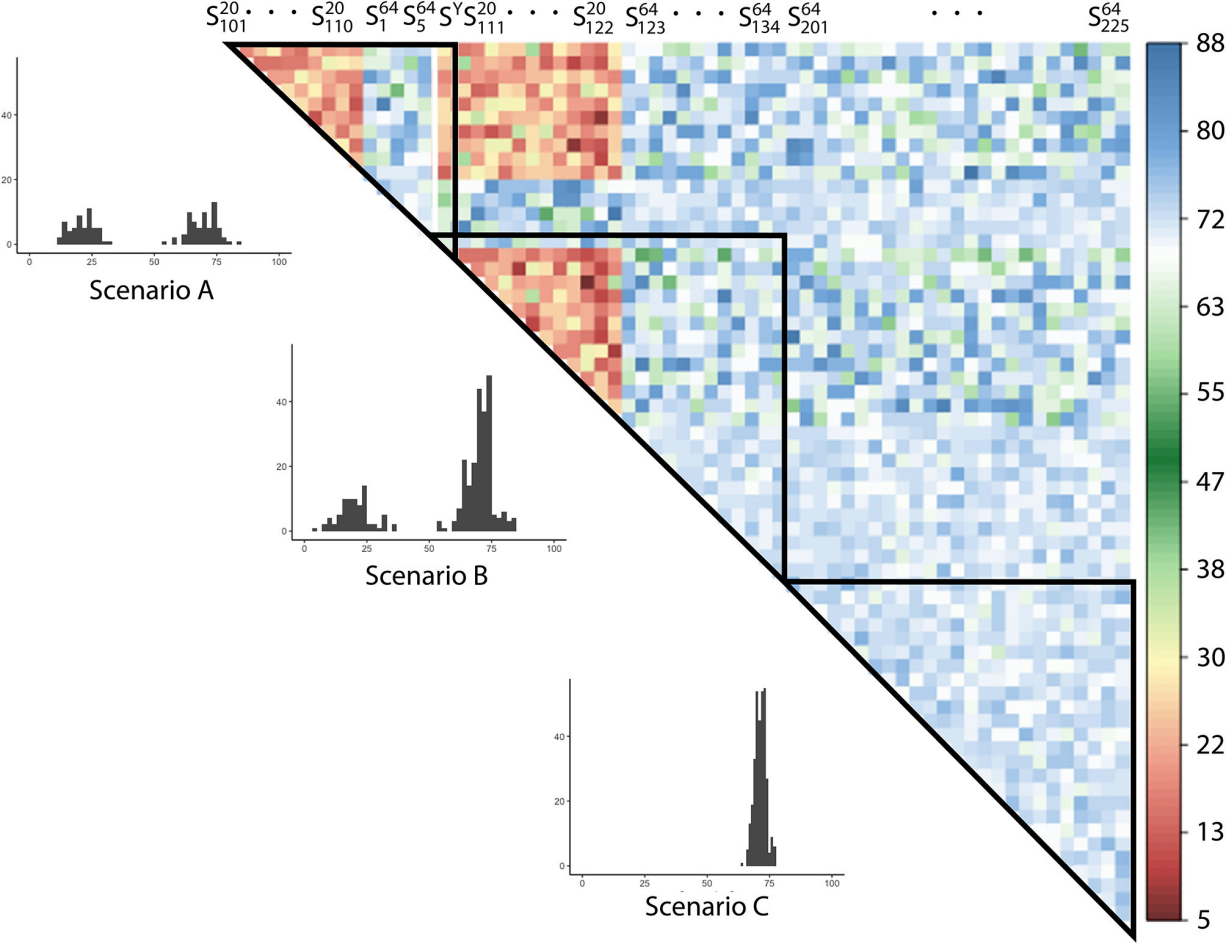
The number of SDS linguistic items overlapping across balanced random subsets. Group-internal overlaps belonging to Scenarios A–C (introduced in section 4.3.2, below) are highlighted in triangles. The histograms show the amounts of overlap for the three scenarios.

The overlap of items across random subsets is visualized in Figure 5. It is visible that the overlap is much smaller (warmer, reddish colors) among *S*^20^ subsets, compared to *S*^64^ subsets, around 20% on average, with some outliers. Overlaps across *S*^20^ and *S*^64^ subsets are much larger (colder colors), with an average of around 70% overlap. The overlaps among *S*^64^ subsets are more uniform (around 70%, with less deviation), as they include by definition all 64 lexical items and the majority of phonetic and morphosyntactic items.

For each *S* subset, we calculate the linguistic matrices. Effects of random item selection in the subsets are shown by Pearson correlation coefficient values across their linguistic matrices, which are almost always above 0.9, with the lowest values around 0.75. The high values (*R*^2^ ≥ 0.62) confirm the similarity across the random subsets even in cases of smaller item overlaps across *S*^20^ subsets.

#### 4.3.1. Clustering and Validation

**Figure 6:**
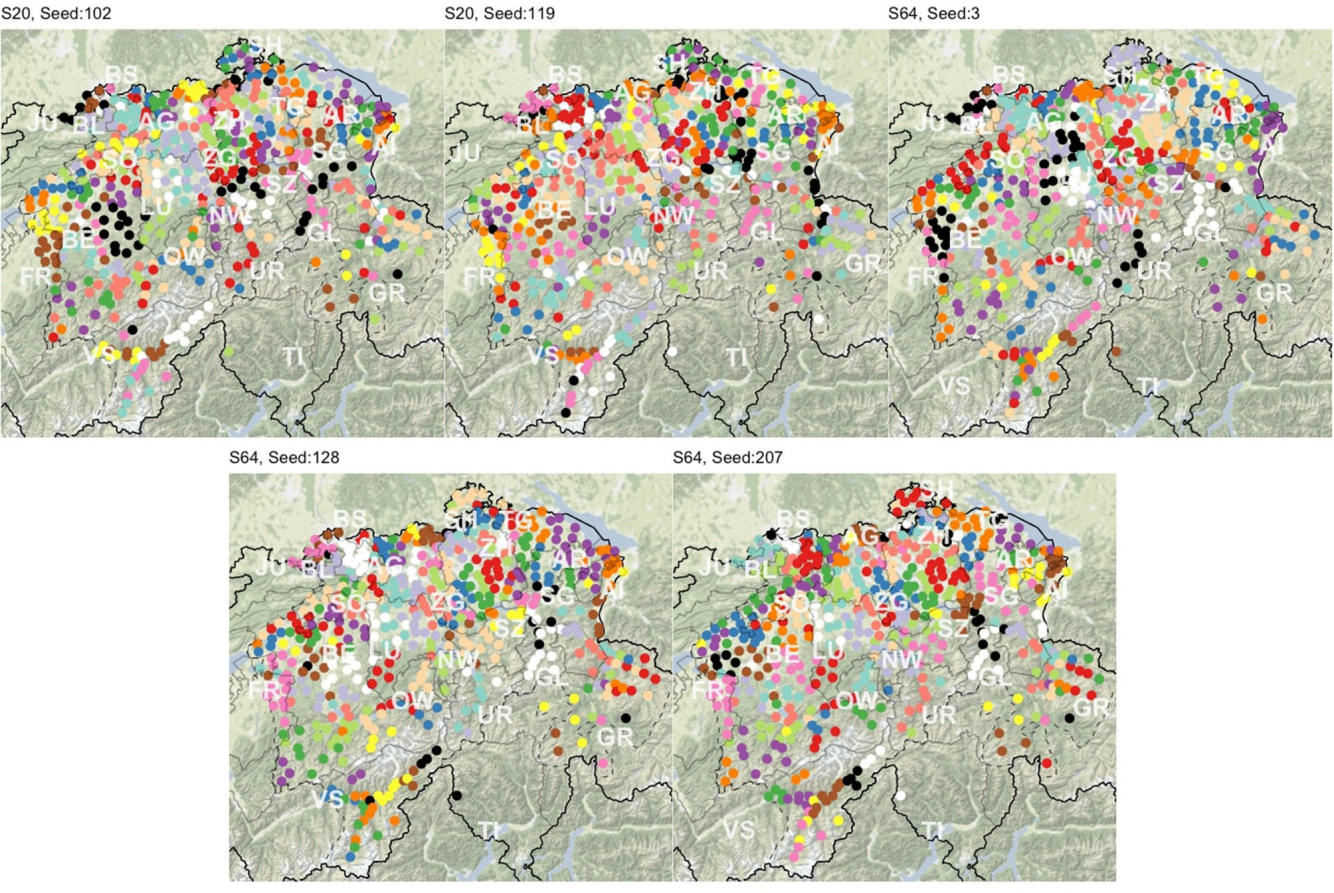
Clusters resulting from five balanced random subsets of SDS variables. Clusters are presented on a diverging scale of 15 colors, which repeat.

We carry out PAM clustering with *k* = 125 on the linguistic distance matrix calculated from each of the *S* subsets, using the cluster package (Maechler et al., 2019) in R. Figure 6 shows the clusters resulting from PAM runs based on variables in five subsets. Structurally, the cluster patterns look similar to the PAM map (Map A) in Figure 2.

To justify using PAM, we test the similarity of *S* subsets' cluster solutions to the cluster solutions of $\bar{LD}$ (PAM, UPGMA, and Ward's method), using Meilă's *VI*. The most important results are shown in Table 2. It becomes visible that PAM clusterings of *S* subsets are more similar to the PAM clustering of the mean linguistic distance matrix ($\bar{LD}$*_PAM*) than the Ward's ($\bar{LD}$*_Ward*) or UPGMA clustering of $\bar{LD}$ ($\bar{LD}$*_UPGMA*). Therefore, if we accept that each of the clustering methods produce linguistically plausible cluster partitions on $\bar{LD}$, then the PAM clustering of the *S* subsets can also be accepted with a high probability.

**Table 2:** Meilă's *VI* values, comparing the cluster partitions across linguistic distance matrices of *S* subsets and cluster partitions of $\bar{LD}$ using the three clustering methods, PAM, UPGMA, and Ward's method.

	**Clustering A**	**Clustering B**	**Meilă's VI**
1	$S_{224}^{64}$ PAM	$\bar{LD}$_PAM	0.3796
2	$S_{126}^{64}$ PAM	$\bar{LD}$_PAM	0.4069
3	$S_{218}^{64}$ PAM	$\bar{LD}$_PAM	0.4096
4	$S_{217}^{64}$ PAM	$\bar{LD}$_PAM	0.4288
5	$S_{4}^{64}$ PAM	$\bar{LD}$_PAM	0.4303
6	$S_{206}^{64}$ PAM	$\bar{LD}$_PAM	0.4370
7	$S_{129}^{64}$ PAM	$\bar{LD}$_PAM	0.4479
8	$S_{211}^{64}$ PAM	$\bar{LD}$_PAM	0.4485
9	$S_{208}^{64}$ PAM	$\bar{LD}$_PAM	0.4509
10	$S_{204}^{64}$ PAM	$\bar{LD}$_PAM	0.4583
...			
46	$S_{128}^{64}$ Ward	$\bar{LD}$_PAM	0.6302
47	$S_{215}^{64}$ Ward	$\bar{LD}$_PAM	0.6398
48	$S_{4}^{64}$ Ward	$\bar{LD}$_PAM	0.6473
49	$\bar{LD}$_Ward	$\bar{LD}$_PAM	0.6476
...			
76	$\bar{LD}$_UPGMA	$\bar{LD}$_PAM	0.7366
77	$S_{221}^{64}$ Ward	$\bar{LD}$_PAM	0.7424
78	$S_{130}^{64}$ UPGMA	$\bar{LD}$_PAM	0.7434
...			
143	phon_Ward	$\bar{LD}$_PAM	0.9909
...			
160	phon_PAM	$\bar{LD}$_PAM	1.0541
...			
255	...	...	...

We validate clusters using a custom method resembling the noisy clustering and bootstrapping approaches often used for cluster validation in dialectometry. For each survey site pair, we note the number of occurrences when the two sites are clustered together. Then, for each survey site, we calculate the percentage (termed *J*) of clustering runs, in which the site is clustered together with the same other survey sites. If survey sites *h*, *i*, and *j* always fall into the same cluster and there is no other survey site ever falling into this cluster, then each of the sites *h*, *i*, and *j* get the maximal *J* value. A survey site that always becomes a singleton would also get this value, giving the chance for very local but unique dialects to stand out as stable clusters.

#### 4.3.2. Selection of Candidate Survey Sites

As in section 4.2.2, we aim to find those survey sites that most affected their surroundings in the last 70 years through the effect of linguistic gravity. To this end, we find the survey site with the highest contemporary population (BFS, 2018) in each cluster, for each of *S* subsets' cluster solution. For each survey site, we note the proportion of *S* subsets' cluster solutions at which the survey site exhibits the highest population in their own cluster. This proportion is termed *P*_*top*_.

**Figure 7:**
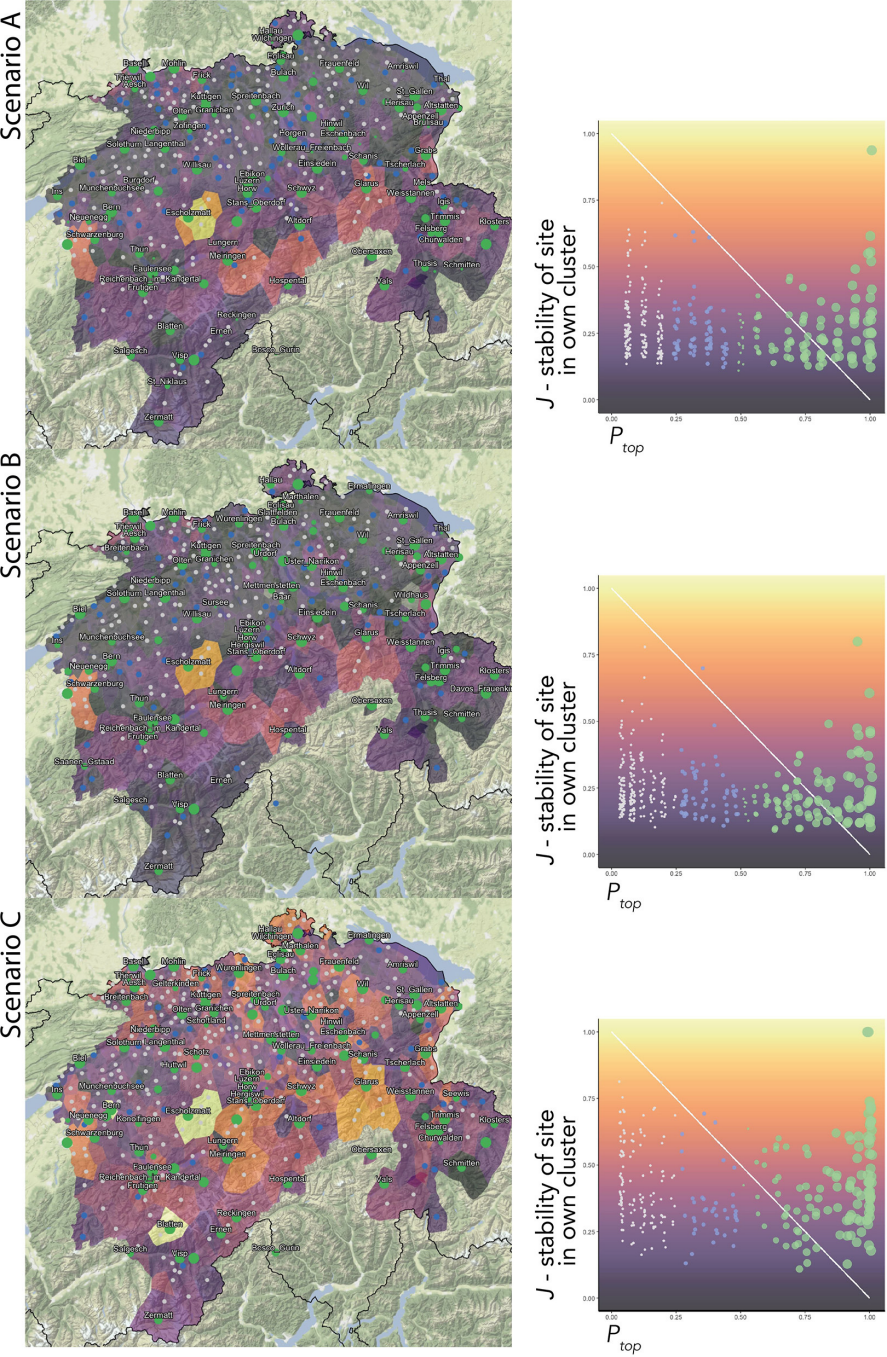
Composite maps and graphs presenting the two candidacy factors deciding the ranking of an SDS survey site to become a candidate site for SDATS. The proportion of occasions a survey site was clustered together with the same others, *J* (stability of a site in its own cluster) is mapped between dark purple and yellow hues, where yellow means higher stability. The number of times (among the clustering solution on different subsets) a site has the most inhabitants in its own cluster is shown by *P*_*top*_. The best candidate sites score high in both *P*_*top*_ and *J*. Such sites are presented as green circles on lighter polygons in the map.

Survey site eligibility is then ranked based on *J* and *P*_*top*_. The scatterplots in Figure 7 show these factors that test the correspondence of SDS sites to SDATS requirements. The *x*-axis, along with the point color and size, presents *P*_*top*_ (green, over 50%; blue, between 25 and 50%; and gray, below 25%, the latter corresponding to a low eligibility for the final SDATS set). The *y*-axis, along with the background color, shows the *J* value of the site, running from dark purple (low “stability in own cluster”) to yellow (high “stability in own cluster”). An ideal survey site would score high with regard to both requirements, reaching the top right corner of the scatterplots. Based on the point color, size, and background color, *J* and *P*_*top*_ can be transferred to the maps on the left. Values of *J* are shown in Maps A–C as the colors of Voronoi-polygons around their SDS sites, illustrating the areal distribution of *J*. Because clusters mostly contain more than one survey site, similar *J* values are expected to cluster in space.

Before we select the candidate sites based on the clustering solutions of all subsets, however, we revisit the potential bias caused by the imbalance across linguistic levels. In the data set, lexical variables make up the smallest portion. However, lexical variables are the most diverse, therefore their variation patterns are potentially the most different from one another and, thus, are associated with greater linguistic distances. Table 3 presents the mean, median, SD, and variance values of the three linguistic levels, with values of the lexical level substantially exceeding the other two levels.

**Table 3:** Descriptive statistics of each linguistic level's linguistic distance matrices.

	**Lexicon**	**Morphosyntax**	**Phonology**
Mean	0.5337	0.4013	0.4001
Median	0.5652	0.4158	0.4216
Standard deviation	0.1771	0.1505	0.1354
Variance	0.0314	0.0226	0.0183

*Besides higher linguistic distance values on average, lexicon has a wider statistical spread as well*.

As we select roughly one-third of the lexical variables in *S*^20^ subsets, there will be a variation in the effect across subsets (as deductible from the overlaps across *S* subsets in Figure 5). *S*^64^ subsets, however, contain all lexical variables, always conveying the full effect of the lexicon.

We aim to select the SDATS survey sites based on a balance across linguistic levels. In order to assess the effect of lexicon, we set up three Scenarios which pool the cluster solutions from a number of *S* subsets. The difference between Scenarios is the proportion to which they contain *S*^64^ subsets, those that convey the full effect of lexicon:

- In Scenario A, the proportion of the full effect of lexicon is 1/3,
- In Scenario B, the proportion of the full effect of lexicon is 1/2,
- Scenario C is entirely made up of *S*^64^ subsets, thus always conveying the full effect of lexicon.

Within the Scenarios, we employ a consensus approach based on the numerous cluster solutions pooled, expecting the cluster solutions, which are somewhat different across subsets, to converge toward a central value. *S*^64^ subsets overlap to a larger degree than *S*^20^ subsets, causing cluster solution across *S*^64^ subsets to be more similar. Therefore, the higher the involvement of *S*^64^ subsets, the higher *J* values are expected. The difference of *J* values across the maps and scatterplots in Figure 7, thus, demonstrates the effect of imbalance across linguistic levels.

With the qualitative revision already taking a foothold in the cluster validation steps, the initial candidate survey sites are selected based on their ranking of *J* and *P*_*top*_. Scenario C's map, regarding the *J* values, resembles the stability map of PAM (Map A) in Figure 2 (despite *J* values not being equivalent to the stability values in a bootstrapping approach), which hints at the similarity of Scenario C and the bootstrap clustering in section 4.2.1. Although our original aim was countering the overweight of lexicon present in $\bar{LD}$, the candidate survey sites resulting from the customized site reduction do not differ substantially across the three Scenarios. This is indicated by the set of survey sites highlighted with their names in Scenario C's map, which forms a superset of sites visible in Maps of Scenario A and B. Thus, it is visible that the qualitative decision of using *P*_*top*_ as a candidacy factor overwrites the effect of linguistic levels. Still, the potential effect of linguistic imbalance is a valid limitation for applications of the general methodology.

Initially, based on the rankings in *J* and *P*_*top*_, 114 candidate survey sites are selected, fewer than the number of clusters originally sought. This is a result of a manual intervention, which is due to the fact that for the aims of SDATS, it is not the number of clusters or their identity that is relevant, but the survey sites' ranking on *J* and *P*_*top*_ values. Beyond the first 114 survey sites, further sites' ranking with regard to either *J* or *P*_*top*_ was too low, thus we decided to leave it to the qualitative revision to fill up the selection, as we ultimately maintain the aim of selecting 125 survey sites.

### 4.4. Revision Based on Linguistic and Sociodemographic Factors

For the qualitative revision of candidate survey sites, we use the candidate site set resulting from section 4.3. This means that the candidate set of survey sites is not equal to the number of clusters sought in the earlier steps. Nevertheless, qualitative revision is not necessarily bound by the clusters or candidate sites yielded by the quantitative steps.

Several reasons impede us from relying fully on the clustering results. First, the cluster partitions reflect the state of the dialectal landscape around 1950, in contrast to the SDATS requirements of investigating contemporary local colloquial dialects. Second, Switzerland has undergone sociodemographic changes, often affecting the composition of the population in settlements. People have become more mobile, and the communities in certain towns and villages recorded in SDS might have changed massively due to industrialization, urbanization, and suburbanization. Third, the digital linguistic data are not entirely optimal for the site reduction. Even if the 289 items in Scherrer's data were representative of SDS, the categorization of the variants within an item corresponds to the needs of SDATS for only 45 items. Besides, if each item is supposed to have the same weight in the process, neither Approach I nor Approach II can completely preclude the disproportionate effect of linguistic levels.

To address these factors, the initial candidate survey site set is evaluated from the following viewpoints, including the indispensable insight of linguists with expertise in past and contemporary dialectal variation. We inspect:

- whether important sociodemographic changes could have occurred at the candidate sites leading to a change or a mixture of dialects. If there was a remarkable change (such as a population boom due to extraordinary economic prosperity, or becoming a touristic hotspot), the location was eliminated from the list of candidate sites (e.g., Uster, canton of Zurich—ZH, or Klosters, canton of Graubünden—GR);
- whether a candidate site's location has merged into another community (e.g., Masans into Chur, GR);
- whether a community is very small or has lost many inhabitants, creating difficulties for recruiting enough respondents from all social backgrounds (e.g., we removed Weisstannen, canton of St. Gallen—SG, but kept Hospental, canton of Uri—UR);
- whether it is known that the local dialect is remarkably interesting (for the general public or from objective linguistics viewpoints). In some cases, local studies have documented change and peculiarities, validating the candidacy of some potential sites (e.g., Bosco Gurin, a partly German-speaking village in the Italian-speaking canton of Ticino—TI, or Blatten, a village in the secluded Lötschental valley in the canton of Valais—VS);
- whether the candidate site is perceived as linguistically representative of the region. For example, the city of Basel is traditionally not regarded as representative of its surrounding region;
- and, less importantly, whether there is a chance for an equidistant choice of survey sites. Following traditional sampling in dialectology, we might select a survey site that makes the survey site set equidistant (counterexamples include candidate sites separated by linguistically significant cantonal borders, e.g., between Niederbipp—BE, and Oensingen, canton of Solothurn—SO).

#### 4.4.1. Revision by Dialectological and Sociogeographic Expertise

Following the aspects listed, the candidate survey sites are revised. The revision is done partly based on sites' rankings on *J* and *P*_*top*_: when a candidate has to be removed, we often turn to these rankings for the next candidate or to validate the choice made based on other factors. For example, Lucerne, Horw, and Ebikon (LU), each were included in the initial set of 114 candidates. However, their geographic proximity allowed them to became a city complex in the last half century, functioning essentially as one unit, with a potential to drawing population from all over Switzerland. Therefore, we only chose Lucerne, the center, to represent this complex. Further, SDS survey sites that have merged in the last 70 years are treated as one, such as Schwamendingen and Zürich (ZH), or Masans and Chur (GR).

Next, we amended the candidate list in a qualitative fashion based on dialect expertise of the SDATS project members. For example, we added locations with local research already present and deemed interesting, such as Jaun (canton of Freiburg—FR), a German-speaking isolate, and Obersaxen (GR), a former island of Walser dialect. We also strived to cover some local dialects deemed peculiar due to the dwindling German-speaking population (Bosco Gurin, TI) or isolation (Vättis, SG). Importantly, major cities and towns of central importance have been taken into the SDATS sample regardless of the clustering results.

At this stage, external dialect experts made further suggestions about available SDS survey sites that are overall representative in their region today, thus not necessarily reflected in the digital data. Some external dialect experts objected to the involvement of urban centers due to the assumptions that urban mixed dialects have already been outliers in SDS, a dialectal phenomenon of rural-urban contrast which needs to be addressed. As we assume dialects of less populated places to converge toward regional hubs, it is indeed beneficial to test this assumption in later analyses with contemporary data if smaller communities are selected along with regional hubs. For example, Reigoldswil (BL), Maur (ZH), and Wilchingen (canton of Schaffhausen—SH) were added for this reason. Additionally, this step has led to dropping some touristic locations assumed to have changed their dialects, such as Klosters (GR), places that became suburbs, such as Pratteln (BL), and to adding more rural varieties, such as Mammern (canton of Thurgau—TG) and Linthal (GL). After consolidating external experts' opinions, the overlap of the initial set of candidate sites and the final selection was 91 out of 114.

**Figure 8:**
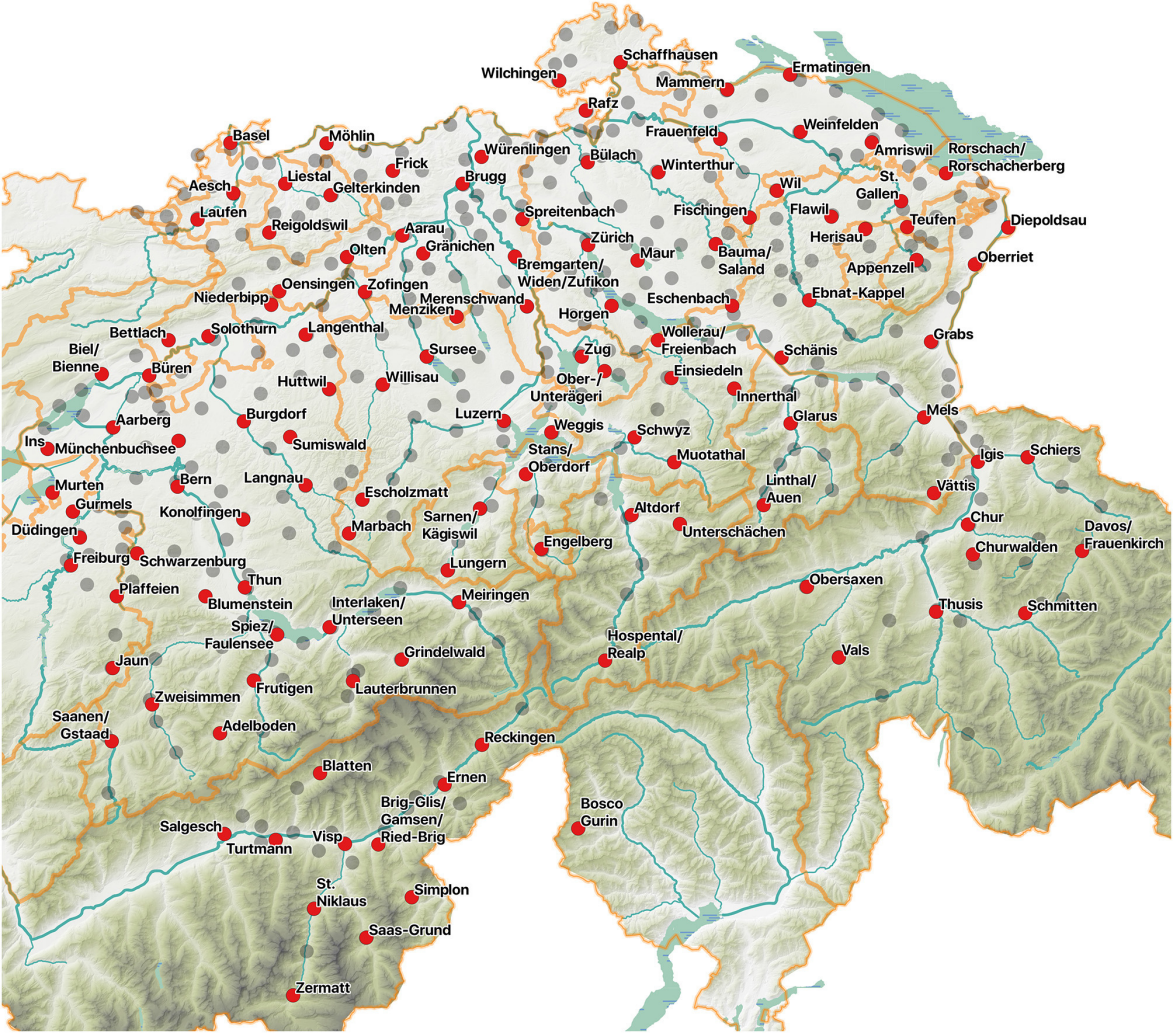
The final selection of SDATS survey sites (in red with names), along with all SDS sites (gray). After the quantitative analysis and the qualitative revision, 125 sites are selected from the original 573.

Finally, Figure 8 presents the conclusive 125 survey sites resulting from the synthesis of the clustering results and their sociodemographic and linguistic revision. In this figure, red sites present those selected for SDATS, while all other SDS survey sites are shown in gray. The distribution of the selected sites is more or less uniform and equidistant, similar to SDS. This means a higher density of SDATS survey sites in the alpine regions, relative to its lower population. At the same time, the alpine region exhibits a greater local variation of dialects, owing to the higher potential isolation caused by more rugged terrain. It has to be noted, however, that the qualitative requirement to have equidistant survey sites did not inform the experiment design.

## 5. Discussion

### 5.1. Summary and Key Findings

Dialectometry uses clustering extensively for determining dialect areas based on linguistic similarity. However, such methods have not been utilized so far for the task of site reduction. We explored this direction with a linguist in mind who aims to revisit dialectal phenomena at representative survey sites of a previously recorded database. We propose a general pathway for incorporating a clustering procedure into the site selection methodology. Since we basically detect clusters in linguistic distance matrices and then appoint a representative survey site in (the spatial projection of) the clusters as candidates subject to a qualitative revision, the methodology is appropriate for several situations. Essentially, the general methodology is based on suggestions and best practices; there is no one-size-fits-all strategy.

Rather than selecting sites based on a grid, we argue for the definition of clusters in non-spatial dimensions, where possible. We demonstrated the quantitative steps of the methodology on data from LAJ as a proof of concept and elaborated a complete application with data from SDS. These examples show that expert revision of candidate survey sites is indispensable, due to the potential bias and uncertainties in the underlying data. Due to constant language change, this expertise appreciates in value with the time elapsed since the collection of the original database.

### 5.2. Interpretation of the Contributions

We find that the following three intertwined aspects impact the choice of specific aspects in the methodology and play a role in the feasibility of an objective implementation:

- An optimal site reduction procedure depends on the overlap of the original and intended studies with regard to their objectives and variables.
- The dialect change that has potentially occurred between the original and intended studies needs to be considered, since the aim of most site reduction tasks is to represent the contemporary dialectal variation.
- Local representation, sought by the applied method, may crucially depend on the purpose of the intended study. Therefore, a qualitative revision of candidate sites may overwrite previous decisions based on several considerations.

By applying the outlined methodology to two databases, we demonstrate that an arbitrary number of representative survey sites can be found within digitized linguistic survey data. The main benefit of the general methodology is to offer candidate survey sites in a quantitative framework, despite the underlying data being potentially fuzzy and uncertain. In a subsequent step, researchers are encouraged to revise the candidate survey sites according to the requirements of their intended study. Specifically, the magnitude of the potential language change that has occurred since the collection of the original data appears to impact the importance of the (partly) qualitative revision over the quantitative steps resulting in candidate survey sites.

The overlap of objectives between the SDS survey and SDATS are decreased by the sociolinguistic aims of SDATS, including SDATS' requirement of more participants per survey site, and its interest in a local colloquial dialect rather than the base dialect. Interpreting the application of the methodology to SDS, we conclude that the individual parameters of the site reduction methodology might be less important than the aims and linguistic knowledge of the researchers. Therefore, the selected 125 survey sites of SDATS are subjective to some degree.

Although not often used in dialectology, we argue that random samples are not ideal for site reduction in a project which specifically aims to capture representative linguistic variation. Even with a high number of random points, following the spatial distribution of the survey genitive sites network, random samples might not follow the distributions in linguistic variables as much as clustering solutions do by design. By selecting the linguistically central points in clusters, one can assert with a higher confidence that the selected site represents the other cluster members.

Our approach essentially also implies that, if digital data are present, it is possible to achieve the representation of the underlying data based on any number of chosen points, e.g., by taking the 20 most distinctive survey sites. This would, of course, lead to a large-scale loss of variation, and it would imply the need for an even more careful qualitative revision after partitioning the data.

### 5.3. Implications for Contemporary Dialectology

The methodology proposed has a number of implications for contemporary research in dialectology and beyond, for sociolinguistics, and more general language surveys. First, the automation of the site reduction process, based on the proposed methodology, allows for greater objectivity in comparison to a traditional approach where researchers have to go through the previous records or atlas data linked to the original survey sites to find the most distinct and/or representative survey sites. The availability of digitized data, clearly, opens opportunities toward faster quantitative approaches.

Second, the usage of cluster validation methods can mitigate the uncertain and fuzzy nature of the underlying data, as reflected in the clustering results. Bootstrapping and noisy clustering methods aid the estimation of this uncertainty during the clustering procedure itself, allowing researchers to adjust their site reduction methods, e.g., the intended number of survey sites, based on stability measures and the aims of their study. Most previous studies (e.g., Kelle, 2001; Christen et al., 2015)^15^ used a grid approach for resampling their respective original set of survey sites and adjusted their sites manually based on expertise.

Third, partitioning clustering algorithms have specific implications. The weakness of partitioning algorithms for the classic usage in dialectometry—finding the *optimal* number of clusters—lies in their sensitivity to outliers. In this regard, however, PAM's *k*-medoid approach is more robust than the *k*-means algorithm, whereas, as seen in the application examples, UPGMA method also seems to produce unreasonable clusters. These clustering algorithms, generally considered successful in dialectology, perform differently on two data sets, suggesting that researchers have to select their methods carefully. Due to the high number of clusters in our case, however, potential outliers often become clusters on their own or together with fellow outliers, regardless of the clustering method. Nerbonne and Wieling (2018) argue for the general usage of hierarchical clustering in dialectometry based on the uncontroversial nature of dialects as hierarchically structured. We argue, however, that at local levels the original hierarchy driven by phylogenetics is not pure, and variation can be more easily overwritten by the radius of local spread of varieties increasing due to the changing contact patterns and increased mobility of the population.

A final benefit of the general methodology is its versatility. As also shown in the application to SDS, survey sites that are outliers in a linguistic sense (e.g., mountain villages in Switzerland, Norway, or Bulgaria), would be uncovered by appropriate clustering in the linguistic space, even if they are spatially embedded in an otherwise homogeneous area (e.g., the Frisian cities in The Netherlands). If the data presents a perfect continuum (e.g., parts of Sweden, as shown by Leinonen, 2010), the application of the methodology with a bootstrapping approach would result in uniformly sized clusters in the abstract linguistic dimensions and in space as well. These clusters, however, would not be very stable, as, due to the continuous nature of the data, specific boundaries between clusters would not be meaningful, and clusters in each bootstrap would be slightly different, without stable “cores.” In such cases, stratifying the area with a uniform spatial grid would also be justified, and a random equidistant survey site network would necessarily represent the variation. The application of the proposed methodology would also be beneficial for smaller studies, aiming to revisit a few phenomena (or new phenomena in a similar linguistic level) in a reduced set of sites. In this case, the low number of variables intentionally overlapping with the aims of the intended study allows for a less biased cluster solution. Further, it is also appropriate to apply the methodology to data other than traditional dialect collections. Sociolinguistic studies, beyond obtaining survey sites in space, could successfully apply the reduction method to quantitatively appoint representative speakers within groups that are identified based on linguistic items and metadata. Moreover, the methodology may help the analysis of contemporary data, such as geotagged tweets collected and then pooled according to some criteria.

## Data Availability Statement

Publicly available datasets were analyzed in this study. These data can be found at: Yves Scherrer's “Sprachatlas der deutschen Schweiz” Digitized dialect maps: http://dialektkarten.ch/mapviewer/swg/index.de.html and at the Linguistic Atlas of Japan Database: https://www.lajdb.org/lajdb_data/LAJDB_data_download001_v20180328_rev.html.

## Author Contributions

The idea, research questions, and conceptualization for this article was developed by PJ, AL, and CS. The data was prepared by PJ and CS. The methodology was worked out and implemented in R and QGIS by PJ. Visualization and data presentation by PJ. The interpretation of results was done by PJ, AL, and CS. The original manuscript was written by PJ, AL, and CS. All authors contributed to the article and approved the submitted version.

## Conflict of Interest

The authors declare that the research was conducted in the absence of any commercial or financial relationships that could be construed as a potential conflict of interest.

## Footnotes

^1^www.sdats.ch

^2^Trüb (2003) mentions that SDS had around 1,500 participants in total, but not the whole questionnaire was answered by all of them. Often the local variety is summarized based on the answers of 2–3 participants answering different parts of the questionnaire (https://sprachatlas.ch/originalmaterial-split/infos).

^3^Available online at the Linguistic Atlas of Japan DataBase (LAJDB)—www.lajdb.org.

^4^For more details on the database and linguistic distance calculation, see Kumagai (2016) and Jeszenszky et al. (2019).

^5^For a visualization of the dialects and linguistic distance in this dataset, including an MDS map, in Japan (without Okinawa), see Jeszenszky et al. (2019, p. 16–18).

^6^By means of e.g., the *silhouette technique* (Rousseeuw, 1987), it is possible to find the optimal number of clusters for partitional clustering, but it is not relevant for most studies in which the present methodology is potentially applicable, as they aim for sampling a higher number of survey sites.

^7^In fpc, the k-medoids clustering is implemented by two algorithms, pam is slower but produces more stable results, while clara is faster but more unstable. For a large number of clusters, clara proved significantly more unstable.

^8^Recently, the LAJ actually received a follow-up at a subset of 554 survey sites (Fukushima, 2016), in the New Linguistic Atlas of Japan (NLJ) (Onishi, 2016). Due to the low number of (only lexical) variables in our data set, however, it would not be sensible to use NLJ as a ground truth.

^9^The digitized data, together with its documentation is available in a tabular format at dialektkarten.ch, where individual variables are also interactively mapped.

^10^For more details, see Jeszenszky et al. (2019, p. 8–9).

^11^Kellerhals (2014) has already produced the MDS maps for each linguistic level, and as an aggregate, based on the contemporary status of Scherrer's data. Also consult Yves Scherrer's homepage, dialektkarten.ch, for average linguistic maps and other parameter maps based on several linguistic atlases.

^12^*Germ.:* Mittelland, the relatively flat part of German-speaking Switzerland from Lake Constance to Lake Bienne in the west.

^13^We use specific *seeds* in R to create reproducible randomized subsets. Setting a seed determines the starting number used to generate a sequence of random numbers; using the same seeds ensures the reproducibility of the same subsets.

^14^Seeds of *S*^20^ and *S*^64^ subsets (shown as the lower indices) do not overlap to avoid complete overlap across the items selected.

^15^A similar study is currently running at the *Deutsch in Österreich* project (DiÖ)—https://dioe.at/projekte/task-cluster-b-variation/pp02/.
